# Ruler elements in chromatin remodelers set nucleosome array spacing and phasing

**DOI:** 10.1038/s41467-021-23015-0

**Published:** 2021-05-28

**Authors:** Elisa Oberbeckmann, Vanessa Niebauer, Shinya Watanabe, Lucas Farnung, Manuela Moldt, Andrea Schmid, Patrick Cramer, Craig L. Peterson, Sebastian Eustermann, Karl-Peter Hopfner, Philipp Korber

**Affiliations:** 1 Division of Molecular Biology, Biomedical Center (BMC), Faculty of Medicine, LMU Munich, Martinsried, Germany; 2 Gene Center, Ludwig-Maximilians-Universität München, Munich, Germany; 3 Department of Biochemistry, Faculty of Chemistry and Pharmacy, Ludwig-Maximilians-Universität München, Munich, Germany; 4 Program of Molecular Medicine, University of Massachusetts, Worcester, MA USA; 5 Department of Molecular Biology, Max Planck Institute for Biophysical Chemistry, Göttingen, Germany; 6 European Molecular Biology Laboratory (EMBL), Structural and Computational Biology Unit, Heidelberg, Germany; 7 Present Address: Department of Molecular Biology, Max Planck Institute for Biophysical Chemistry, Göttingen, Germany; 8 Present Address: Department of Cell Biology, Blavatnik Institute, Harvard Medical School, Boston, USA

**Keywords:** DNA, Enzyme mechanisms, Chromatin remodelling, Chromatin structure, Nucleosomes

## Abstract

Arrays of regularly spaced nucleosomes dominate chromatin and are often phased by alignment to reference sites like active promoters. How the distances between nucleosomes (spacing), and between phasing sites and nucleosomes are determined remains unclear, and specifically, how ATP-dependent chromatin remodelers impact these features. Here, we used genome-wide reconstitution to probe how *Saccharomyces cerevisiae* ATP-dependent remodelers generate phased arrays of regularly spaced nucleosomes. We find that remodelers bear a functional element named the ‘ruler’ that determines spacing and phasing in a remodeler-specific way. We use structure-based mutagenesis to identify and tune the ruler element residing in the Nhp10 and Arp8 modules of the INO80 remodeler complex. Generally, we propose that a remodeler ruler regulates nucleosome sliding direction bias in response to (epi)genetic information. This finally conceptualizes how remodeler-mediated nucleosome dynamics determine stable steady-state nucleosome positioning relative to other nucleosomes, DNA bound factors, DNA ends and DNA sequence elements.

## Abstract (web summary)

Although chromatin remodelers have been shown to align nucleosome arrays to barriers and to generate spacing regularity among nucleosomes within arrays, it has remained unclear how the distance to barrier and the spacing length are determined in absolute terms. Here, the authors reveal that remodelers contain a ‘ruler’ element that sets remodeler-specific alignment and spacing distances when generating nucleosome arrays.

## Introduction

Nuclear DNA is packaged into chromatin based on a repeating building block, the nucleosome core particle (NCP^1,2^), where 147 base pairs (bp) of DNA are wound around a histone protein octamer^3^. Packaging by NCPs orchestrates all genomic processes^4^.

NCPs mainly occur in regular arrays^5^ where they are aligned to each other such that the lengths of linker DNA between NCPs are about constant within an array. An NCP plus linker constitutes the nucleosome, although this term is often used for an NCP by itself, too. Linker lengths may vary among arrays in the same cell^6–9^ and their cell averages differ between cell types and species^10^. Arrays are often phased, i.e., aligned relative to a genomic reference point. A combination of both in vivo studies^11–17^ and in vitro reconstitutions^18^ indicated that these genomic alignment points or “barriers” often reflect the binding of abundant, sequence-specific DNA binding proteins, like the general regulatory factor (GRFs) Reb1, Abf1, or Rap1 in budding yeast or other architectural factors like CTCF in mammals^19^ or Phaser in flies^20^.

Throughout eukaryotes, phased arrays are prominent at active promoters. Nucleosome-depleted regions (NDRs) at the core promoter are flanked by arrays that begin with the so called +1 nucleosome close to the transcription start site (TSS) and cover the gene body^4,5^. This organization is important for transcription fidelity as mutants with impaired array phasing show aberrant transcription initiation^21–26^. While nucleosome arrays are likely the most pervasive and longest known chromatin organization, their generation is still not explained. Specifically, regular spacing requires fixed distances between nucleosomes, and phasing requires a fixed distance between array and reference point. What sets these distances?

In vivo and in vitro data suggest that ATP-dependent chromatin remodeling enzymes (remodelers) are key to the answer. Remodelers are conserved in eukaryotes^27^ and mobilize, reconfigure, or disassemble/reassemble nucleosomes upon ATP hydrolysis^28,29^. They are subdivided into the SWI/SNF, ISWI, CHD, and INO80 families, according to their main ATPase sequence features. Besides the core ATPase, remodelers often contain additional domains and subunits that bind the nucleosome, regulate activity and targeting, and convert their DNA tracking activity into the remodeler-specific chemo-mechanical reaction. For example, nucleosome disassembly is accomplished only by SWI/SNF family members and histone exchange only by INO80 family members, while nucleosome sliding is catalyzed by most remodelers.

Particularly relevant for array generation is an ATP-dependent nucleosome spacing activity, by which some remodelers convert irregular arrays into arrays of regularly spaced nucleosomes. Remodelers of the ISWI, CHD, and INO80^30–33^, but not of the SWI/SNF family, show spacing activity. This activity was suggested to rely on a length-sensor mechanism^34,35^ where nucleosome sliding rate is regulated by linker DNA length. Sliding one nucleosome back and forth between two other nucleosomes, with a linker length-dependent velocity, would center a nucleosome at steady state when both flanking linkers have the same length.

While the length-sensor mechanism may equalize linker lengths and thereby generate spacing distance regularity, it does not by itself determine spacing distance length in absolute terms. This would reciprocally depend on nucleosome density. However, spacing in vivo^13,23,36^, as well as generated in vitro^37,38^, remained constant despite changes in nucleosome density. This was called “active packing”^38^ or “clamping”^37^, but it remained unclear if remodeler or nucleosome features led to such density-independent spacing.

Structural studies suggested that the yeast ISW1a remodeler contacts a neighboring nucleosome and may set the linker length by a “protein ruler”^39^. Two ISWI family remodelers, yeast ISW1a and ISW2, each generated regular arrays aligned at DNA-bound Reb1 or Abf1 in vitro, but with different spacing at the same nucleosome density^18^. This points toward a remodeler-specific linker length determining ruler mechanism. Also suggestive of a built-in ruler, INO80 required a minimum linker length for nucleosome sliding^35^ and recognized linker DNA via a structural module that was important for sliding^40^.

The ruler metaphor may indeed describe a remodeler mechanism that measures and sets the phasing and spacing distances of arrays. However, so far it is mainly suggestive and has to be substantiated in molecular terms. This would be exceedingly convoluted in vivo but requires a defined system that allows to assay the generation of phased regular arrays by remodelers and to dissect if and how a ruler mechanism is at work. Are there rulers within some or all remodelers with spacing activity? Are linker length vs. distance to barrier determined in the same or different way? Are rulers autonomous or does the outcome depend on nucleosome density or underlying DNA sequence? Ultimately, is it possible to tune a ruler, i.e., can a remodeler be mutated to generate arrays with altered spacing and/or phasing distances?

Here, we used genome-wide in vitro chromatin reconstitution with purified remodelers (ref.  ^18^, accompanying paper^41^) to answer these questions. All yeast remodelers with known spacing activity, ISW1a, ISW2, Chd1, and INO80 have rulers that are largely autonomous regarding underlying DNA sequence but some may respond to nucleosome density. Remodeler-specific rulers mechanistically explain earlier in vivo observations. Structure-guided mutations in recombinant INO80 complexes led to shorter or longer spacing and phasing distances and showed that these quantities may be uncoupled. Finally, we propose a model how remodeler rulers position nucleosomes by regulating sliding direction bias according to (epi)genetic information in the nucleosome environment.

## Results

### Defined genome-wide chromatin reconstitution system with varying nucleosome densities

**Fig. 1:**
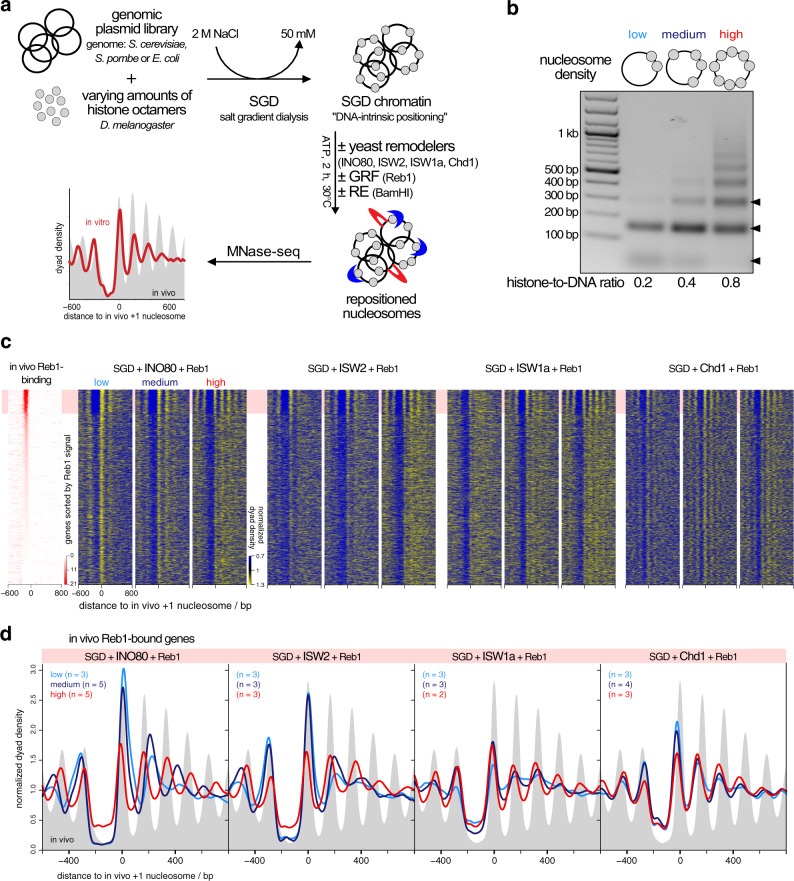
Reb1-guided nucleosome positioning in vitro by individual remodelers at varying nucleosome density. **a** Overview of genome-wide in vitro reconstitution system. (GRF general regulatory factor, RE restriction enzyme). **b** Comparison of SGD (salt gradient dialysis) chromatin reconstituted at indicated histone-to-DNA mass ratios. DNA fragments after Micrococcal Nuclease-digest under the same conditions were resolved by agarose gel electrophoresis. Arrow heads on right point to subnucleosomal, mono- and dinucleosomal fragments (bottom to top). MNase digest and agarose gel electrophoresis was performed for every SGD chromatin sample. Results were similar to those shown here. **c** Heat maps of MNase-seq data for SGD chromatin of the indicated nucleosome density (low, medium, high) and after incubation with indicated remodelers and Reb1. Chd1 refers to the Chd1/FACT complex. Heat maps are aligned at in vivo +1 nucleosome positions and sorted according to decreasing (top to bottom) anti-Reb1 SLIM-ChIP score^49^ (Reb1 signal) as measure for in vivo Reb1 binding and shown in leftmost heat map. Horizontal red shading highlights genes with strong in vivo Reb1 binding at promoters. Merged data of replicates are shown, individual replicates in Supplementary Fig. 1b, c. **d** Composite plots for data as in **c** averaged over the indicated number of replicates (*n*) and only for genes highlighted in red in **c**. Gray background shows in vivo MNase-seq data generated in this study.

To assess array generation by remodelers in a biochemically defined way, we used our genome-wide chromatin reconstitution system with purified components (Fig. 1a^18^) including recombinant INO80 complex (accompanying paper^41^) and recombinant Chd1^42^. Briefly, genomic plasmid libraries were reconstituted with *Drosophila* embryo histone octamers into nucleosomes by salt gradient dialysis (SGD). SGD chromatin was incubated with ATP, purified yeast remodelers (Supplementary Fig. 1a), and the barrier Reb1 or the restriction enzyme BamHI, which generates double strand breaks (DSBs) that also amount to nucleosome positioning barriers (accompanying paper^41^). Resulting nucleosome patterns were analyzed by MNase-seq. The effective histone-to-DNA mass ratio during SGD was varied from 0.2 to 0.8 yielding low, medium, and high nucleosome densities reflected in increasingly extensive MNase-ladders at the same MNase digestion conditions (Fig. 1b). Nucleosome density variation was instrumental to distinguish if linker lengths and phasing distances depended on nucleosome density and/or remodeler features.

### INO80, ISW2, ISW1a, and Chd1, but not Fun30 align regular arrays at the barrier Reb1

We tested all yeast remodelers with known spacing activity, INO80, ISW2, ISW1a, and Chd1^18,32,33,43–45^ as well as the Fun30 remodeler, for which it was unclear if it has spacing activity^46^. INO80, ISW2, ISW1a, and Chd1, each in combination with Reb1, generated phased regular arrays at promoters with Reb1 sites (red shaded top of heat maps in Fig. 1c), while Fun30 did not (Supplementary Fig. 1b). This clarifies that Fun30 does not have regular array generation and alignment activity.

Previously, Chd1 purified from budding yeast did not show much effect in genome-wide reconstitutions^18^. This was maybe due to full-length Chd1 tending to aggregate in vitro, which is why truncated Chd1 constructs were often used^47,48^. Here, we leveraged our finding that recombinant full-length Chd1 is stabilized in complex with recombinant FACT complex^42^ and achieved in vitro array generation and alignment also by Chd1.

The heat map patterns (Fig. 1c) and even more the corresponding composite plots for the Reb1-bound genes only (Fig. 1d) suggested that the distance of arrays to the barrier Reb1 as well as the linker lengths varied with nucleosome density in a remodeler-specific way. For all remodelers with spacing activity, array extent increased with growing density, consistent with greater nucleosome availability and processive spacing activity. Array extent at high density was larger than in our previous reconstitutions^18^, i.e., we achieved higher densities here. Adding more remodeler after half of the incubation time did not change the array distances of resulting patterns confirming non-limiting remodeling activity and steady-state conditions (Supplementary Fig. 1c). The observation that nucleosome phasing and spacing was largely invariant for Chd1 samples at all densities excluded that respective differences for the other remodelers were due to MNase digestion degrees varying with nucleosome densities.

### Remodelers set phasing and spacing distances symmetrically around barriers

**Fig. 2:**
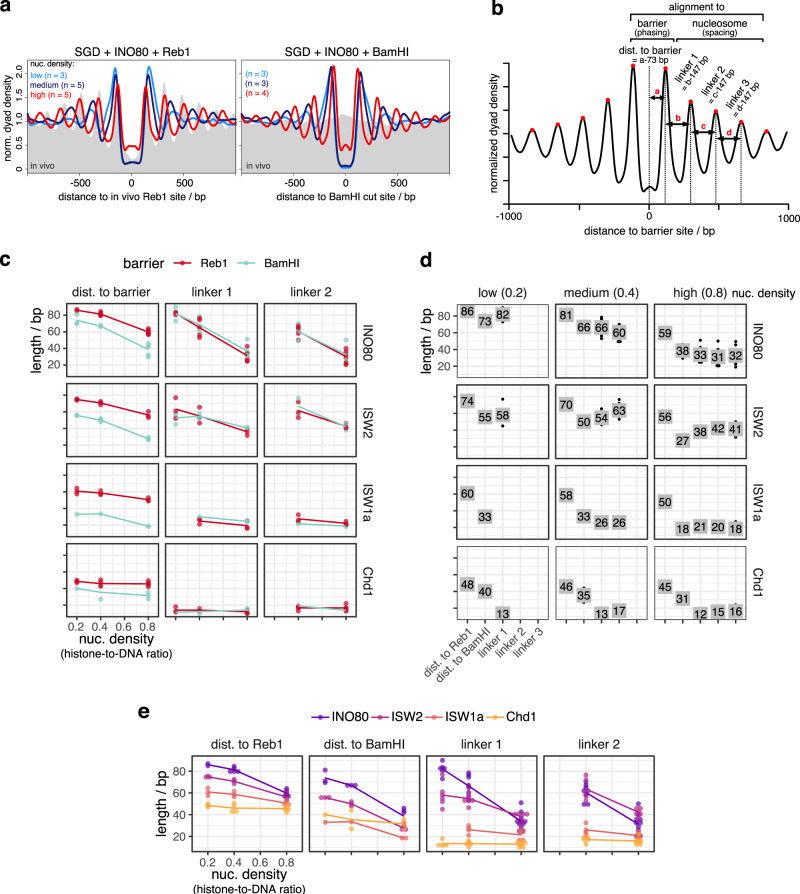
Quantification of how barrier-aligned nucleosome array features depend on barrier type, remodeler type, and nucleosome density. **a** Composite plots of same MNase-seq data for INO80 and in vivo as in Fig. 1d but aligned at in vivo Reb1 sites (left), or at BamHI sites (right) of SGD chromatin reconstituted at the indicated nucleosome densities and incubated with INO80 and BamHI. **b** Scheme defining array features quantified for barrier-aligned composite plots as in **a**. Array feature values for the indicated combinations of barrier, remodeler, and nucleosome density plotted in different ways allowing comparison between barriers (especially (**c**)), values (especially (**d**)), and remodelers (especially (**e**)). Chd1 refers to the Chd1/FACT complex. Every dot in **c**–**e** represents one replicate; the lines in **c** and **e** and the numbers in **d** indicate the mean. Replicate dots are often hidden underneath numbered box in **d**. Composite plots of individual replicates for data shown in **a**, **c**–**e** in Supplementary Fig. 2c.

To better assess distances to barrier (phasing) and linker lengths (spacing), we aligned the MNase-seq data for each remodeler/barrier/density combination to either in vivo Reb1 sites or BamHI sites (Fig. 2a). We chose Reb1 sites called in vivo by SLIM-ChIP^49^ as we saw strong effects upon alignment at these sites in contrast to sites called for Reb1 binding to free DNA in vitro by PB-exo^50^ only (Supplementary Fig. 2a, b). For each replicate (Supplementary Fig. 2c–e), we called nucleosome peaks in the composite plot and determined the distances to barrier and linker lengths as defined in Fig. 2b.

All remodelers symmetrically aligned regular arrays to BamHI sites, which are palindromic and therefore inherently symmetrical, and most of them also to Reb1 sites (Fig. 2a and Supplementary Fig. 2c, d) regardless of site orientation and position relative to genes (groups 1–3; Supplementary Fig. 3a, b). However, if INO80 aligned arrays at promoter Reb1 sites (groups 1–3, Supplementary Fig. 3a, accompanying paper^41^), nucleosome occupancy (peak height) was higher over genic vs. non-genic regions at low and medium nucleosome density leading to asymmetric patterns with regard to peak heights in groups 1 and 2. Reb1 site orientation had no effect (group 1 vs. 2). This asymmetry in nucleosome occupancies reflected that positioning of +1 nucleosomes, per definition the first nucleosomes downstream of TSS, was not only guided by Reb1 bound to promoter sites but also synergistically by underlying DNA shape/mechanics features (accompanying paper^41^). We recapitulated here that INO80 was able to position in vivo-like +1 nucleosomes in the absence of a barrier at low and medium densities (Supplementary Fig. 3c, d). This synergism between Reb1- and DNA shape/mechanics-guided +1 positioning at low and medium density resulted in higher occupancy at the +1 nucleosomes, which are alignment points for +2 nucleosomes and so on. Therefore, all array peaks over genes were higher than their counterparts over non-genic regions at these densities.

However, such synergism was not seen at high density where in vivo-like +1 nucleosome positioning by INO80 alone was much less pronounced (Supplementary Fig. 3c, d). The latter was not due to a general inability of INO80 to slide densely packed nucleosomes as INO80 could generate Reb1- or DSB-aligned arrays at these high nucleosome densities, too (Figs. 1c, d and 2a and Supplementary Fig. 2c, d). Nonetheless, at high nucleosome density, both this alignment activity and the spacing activity without Reb1 were apparently incompatible with or dominant over DNA shape/mechanics-guided nucleosome positioning (see “Discussion”). This showed again that our here generated high nucleosome density was higher than the nucleosome density used previously^18^, otherwise in vivo-like +1 nucleosome positioning by INO80 would not have been clearly observed in our earlier study. In this context, we also tested if Fun30 positions in vivo-like +1/−1 nucleosomes on its own, but it did not (Supplementary Fig. 3d).

In contrast to nucleosome peak heights, nucleosome peak positions and therefore corresponding phasing and spacing distances were not significantly affected across groups 1–3 for all remodelers, including INO80 (Supplementary Fig. 3b). Therefore, all remodelers symmetrically generated phasing and spacing distances at Reb1 and BamHI sites, which warranted averaging over the up- and downstream values. Resulting values were plotted in different ways to facilitate multi-dimensional comparisons (Fig. 2c–e). As all remodelers generated linker lengths independently of the barrier type, we combined linker length values for both barriers (Reb1 and BamHI, Fig. 2c). Linker length determination relied on nucleosome peak calling in composite plots, which was often not possible beyond the −1/+1 nucleosomes at low nucleosome density (Supplementary Fig. 2c), so that linker length data for low-density conditions were more sparse, for ISW1a even absent.

We note that the up- and downstream distances of flanking nucleosomes to Reb1 in vivo differed at unidirectional promoters with ca. 60 bp vs. ca. 80 bp, respectively (Supplementary Fig. 3b, groups 1 and 2), which averages out to ca. 70 bp at bidirectional promoters (group 3) and at all, not just promoter, Reb1-sites (“all sites”) as the up- and downstream distinction of transcription directionality is lost in both latter cases. We assume that this asymmetry is linked to the synergistic determination of +1 nucleosome positions by both Reb1 and DNA shape/mechanics features (accompanying paper^41^) and/or additional constraints at promoters.

### Remodeler-specific rulers set spacing in a density-independent or -dependent way

To compare spacing generated by different remodelers at different nucleosome densities, we focused on the averaged length of linker 1 (Fig. 2b), which was most accessible across all nucleosome densities. Chd1 generated the shortest (12–13 bp) and ISW1a a bit longer (21–26 bp) linker 1 lengths without significant effects by nucleosome density (Fig. 2c–e). ISW2 generated rather constant spacing (54–58 bp) at low and medium but tighter spacing (38 bp) at high density. For INO80, linker lengths steadily increased with decreasing density from 33 to 82 bp.

We concluded that linker lengths and their dependencies on nucleosome density were remodeler-specific and interpreted this as follows. Spacing activity of a remodeler has two aspects. On the one hand, the remodeler equalizes linker lengths leading to regularity in arrays, which is the classical definition of spacing activity^30,31^. On the other hand, the resulting linkers have a certain length. In our purified system, this may either be determined by nucleosome density and/or by a remodeler-intrinsic feature. Following Yamada et al.^39^, we call a remodeler feature that sets nucleosome spacing a “ruler”. We use this term also for the feature that sets the distance to barriers (see below). Indicative for a remodeler ruler is remodeler-specific clamping, i.e., if constant spacing is generated at different nucleosome densities (= clamping) and different remodelers generate different spacing (= remodeler-specific), which shows that spacing depends on remodeler-intrinsic and not nucleosome-intrinsic properties^37^. We saw remodeler-specific clamping for Chd1 at all, for ISW1a at high vs. medium and for ISW2 at medium vs. low densities (Fig. 2c–e). This argues for rulers in these remodelers, but does not preclude additional density effects. As none of the remodelers with spacing activity can disassemble nucleosomes^29^ and thereby affect nucleosome density, their rulers can only set their full respective linker lengths if these are shorter than or equal to the density-determined linker length at equidistant nucleosome distribution. Accordingly, Chd1 and ISW1a set their full ruler-specified linker lengths at all and ISW2 at medium and low densities. ISW2 had to generate shorter linkers at high density. INO80 either did not have a ruler or the ruler responded to changes in nucleosome density.

In vitro mononucleosome assays suggested that INO80 requires at least 40 bp of nucleosome-free DNA for nucleosome sliding^35^, while it generated 30 bp linkers in trinucleosomes^33^. Here, at high nucleosome density, INO80 generated linkers of about 33 bp consistent with previous observations. We tried to enforce even tighter spacing by increasing nucleosome density. This did not decrease spacing and phasing distances but peak heights (Supplementary Fig. 2d, e), probably due to increased aggregation without effective increase in nucleosome density of soluble chromatin.

### Remodeler type, barrier type, and nucleosome density determine distance to barrier

The findings for the distance to barrier were more complex than for lengths of linker 1 (Fig. 2c–e). First, the distance to barrier depended on the barrier type (Fig. 2c). It was always longer for Reb1 than for BamHI generated DNA ends, with the largest difference for ISW1a and the smallest for Chd1. The DNA footprint size of *S. cerevisiae* Reb1 is not known, possibly 20 bp as for the *S. pombe* Reb1 DNA binding domain (DBD)^51^. This would contribute ~10 bp to the distance to barrier (Fig. 2b) and could explain the differences between distance to Reb1 vs. BamHI sites for Chd1, but not for the other remodelers. Therefore, INO80, ISW2, and ISW1a, but not Chd1, aligned nucleosomes differently at Reb1 vs. at DSBs.

Second, the distance to DNA ends was mostly similar to linker lengths for INO80, ISW2, and ISW1a, arguing that these remodelers, but not Chd1, used a DNA end in a similar way as a neighboring nucleosome for nucleosome alignment.

Third, distances to barriers were (in)dependent on nucleosome density in a similar way as linker lengths for all remodelers but INO80, where distances to both barriers varied less between low and medium density than linker length.

We concluded that there are remodeler-specific differences in how a nucleosome is positioned next to another nucleosome vs. next to a barrier like Reb1 vs. next to a DNA end and how this depends on nucleosome density. This is again a clear case of different remodelers generating different nucleosome positioning, although starting from the same SGD chromatin, which argues for remodeler-specific rulers governing nucleosome positioning.

### Remodelers differ in processivity of nucleosome positioning

All remodelers generated similar lengths of linker 1 to linker 3 at high density (Fig. 2d), which we interpreted as processive spacing activity along the arrays as long as nucleosomes were sufficiently provided. At low density, ISW2, Chd1, and especially INO80 still generated high +1/−1 nucleosome peaks (Supplementary Fig. 2c), in contrast to ISW1a, for which these peaks were less pronounced and +2/−2 nucleosome peaks could not be discerned. We suggest that ISW1a is less processive than other remodelers in bringing nucleosomes next to barriers or other nucleosomes at low densities.

### Remodelers generate similar arrays on all but more effectively on eukaryotic DNA sequences

**Fig. 3:**
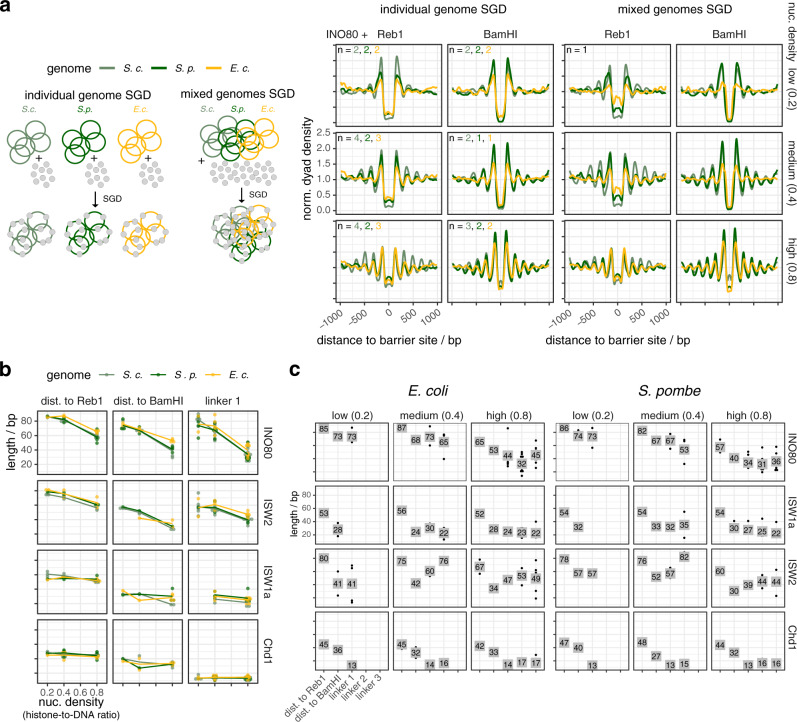
Yeast remodelers generate arrays on heterologous genomes with same spacing and phasing distances as on the yeast genome. **a** Left: schematic similar to Fig. 1a showing SGD reconstitution with individual or mixed genomes. Right: composite plots of merged replicates as Fig. 2a but for the indicated barriers and either individual (left) or mixed (right, one replicate for each condition) genomes. Composite plots of individual replicates in Supplementary Fig. 4a. Reb1 sites were called by PWM. **b**, **c** As Fig. 2c, d, respectively, but for the indicated genomes.

The same linker lengths in arrays at BamHI and Reb1 sites (Fig. 2c), at Reb1 sites in groups 1–3 and the symmetry of nucleosome distances to Reb1 sites in groups 1–3 (Supplementary Fig. 3a, b) suggested that remodeler rulers position nucleosomes independently of DNA sequence flanking the barriers. Nonetheless, there are evolved DNA features at promoters, especially recognized by INO80 (accompanying paper^41^), that affected occupancies (peak heights, not positions, Supplementary Fig. 3a), which may also be true for evolved nucleosome-favoring dinucleotide periodicities^52^ in gene bodies. To rigorously disentangle these contributions, we tested the remodeler/barrier/density combinations also with SGD chromatin of *S. pombe* and *E. coli* genomic plasmid libraries (Figs. 1a and 3a–c and Supplementary Fig. 4a), including the steady-state control (Supplementary Fig. 4b). We did not observe substantial differences in spacing/phasing distances on these genomes for all remodelers, but some replicates, especially at medium and low density, showed lower relative nucleosome occupancies for the *E. coli* genome.

We concluded that all remodelers align arrays at Reb1 or DSBs regardless of the underlying sequence. Nonetheless, they are more effective in terms of relative occupancies on eukaryotic genomes, likely due to dinucleotide periodicities^53^.

### INO80 complexes mutated in the Arp8 and/or Nhp10 module

It was unexpected that the clamping criterion did not clearly show a ruler for INO80 (Fig. 2c–e), because the INO80 structure suggested modules that bind extranucleosomal DNA and could serve as ruler^40^. To clarify, we took advantage of the biochemical accessibility of our recombinant INO80 preparation, the modular INO80 composition, and the high resolution structures^40,54^ to generate candidate mutations that may tune and thereby reveal INO80’s ruler.

**Fig. 4:**
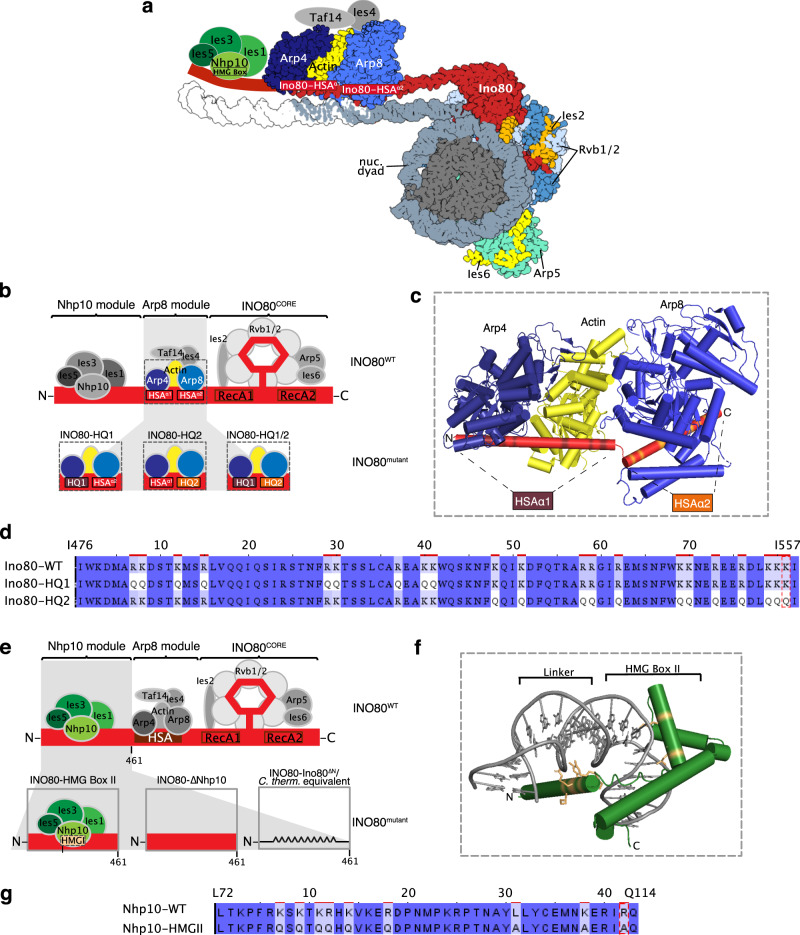
Construction of INO80 mutant complexes. **a** Structure-based^40,54^ model of a nucleosome bound by the INO80 complex with indicated subunits. Nhp10 module, Taf14, and Ies4 organization is assumed. **b** Schematic of INO80 complex submodule and subunit organization (top). Zoom into Arp8 module showing three mutant versions (bottom). **c** Cylindrical representation of the Arp8 module structure showing mutated residues of Ino80-HSA domain (highlighted in brown and orange). **d** Sequence alignment showing mutated residues in Ino80-HQ1 and -HQ2 mutants. **e** Schematic of INO80 complex organization as in **b** (top) but zoom into Nhp10 module (bottom) showing three mutant versions. **f** Model of Nhp10 HMG box-like and Linker region (residues 62–172) based on TFAM structure (pdb 3tq6). **g** Sequence alignment showing mutated residues in Nhp10-HMGII mutant. Images of **a**, **b**, **e**–**g** are also shown in the accompanying paper^41^. Color code for subunits is consistent across panels.

The INO80 complex has two modules with a likely role in ruler function. First, the Arp8 module consisting of N-Actin, Arp8, Arp4, Taf14, and Ies4 (Fig. 4a). It binds the Ino80 main ATPase HSA domain, which is structured as a long helix with a kink that subdivides it into the HSAα1 and HSAα2 part^40^. Both bind to extranucleosomal DNA, and mutating DNA contacting lysine residues in HSAα1 or HSAα2 to glutamines (HQ1 and HQ2 mutant, respectively, Fig. 4b–d) impaired, and combining both mutations (HQ1/2 mutant) abolished mononucleosome centering activity^40^.

The second, Nhp10 module, binds the Ino80 ATPase N-terminus, and contains the HMG box Nhp10 subunit, along with Ies1, Ies3, and Ies5 (Fig. 4a, e). This module is species-specific and affects the processivity and extranucleosomal DNA requirements in mononucleosome sliding assays^35^. Calculating a homology model for Nhp10 based on another HMG box protein, TFAM^55^, we inferred and mutated amino acid residues putatively involved in Nhp10-DNA interactions (HMGII mutant, Fig. 4f, g). These mutations were also combined with the HQ1 or HQ2 mutants (HMGII-HQ1 and HMGII-HQ2). Further, we prepared recombinant INO80 complex without any Nhp10 module subunits (ΔNhp10 mutant, no truncation of the Ino80 ATPase N-terminus) or a version where the Ino80 ATPase lacked residues 1–461 (INO80-Ino80^ΔN^ mutant), which removes the assembly platform for the Nhp10 module (Fig. 4a, e).

### Mutant INO80 complexes reveal a multilayered ruler

**Fig. 5:**
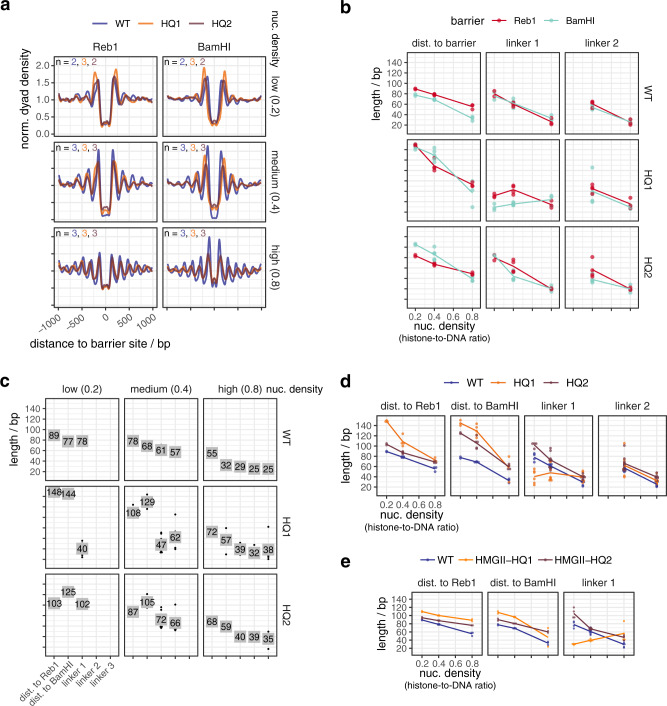
Mutations in the INO80 Arp8 module affect the generation of array features. **a** Composite plots of merged replicates as in Fig. 2a but for the indicated WT and mutant INO80 complexes and nucleosome densities. Composite plots of individual replicates in Supplementary Fig. 5a. **b**–**d** As Fig. 2c–e, respectively, but comparing indicated WT and mutant INO80 complexes. **e** As Fig. 2e, but for the indicated WT and mutant INO80 complexes.

**Fig. 6:**
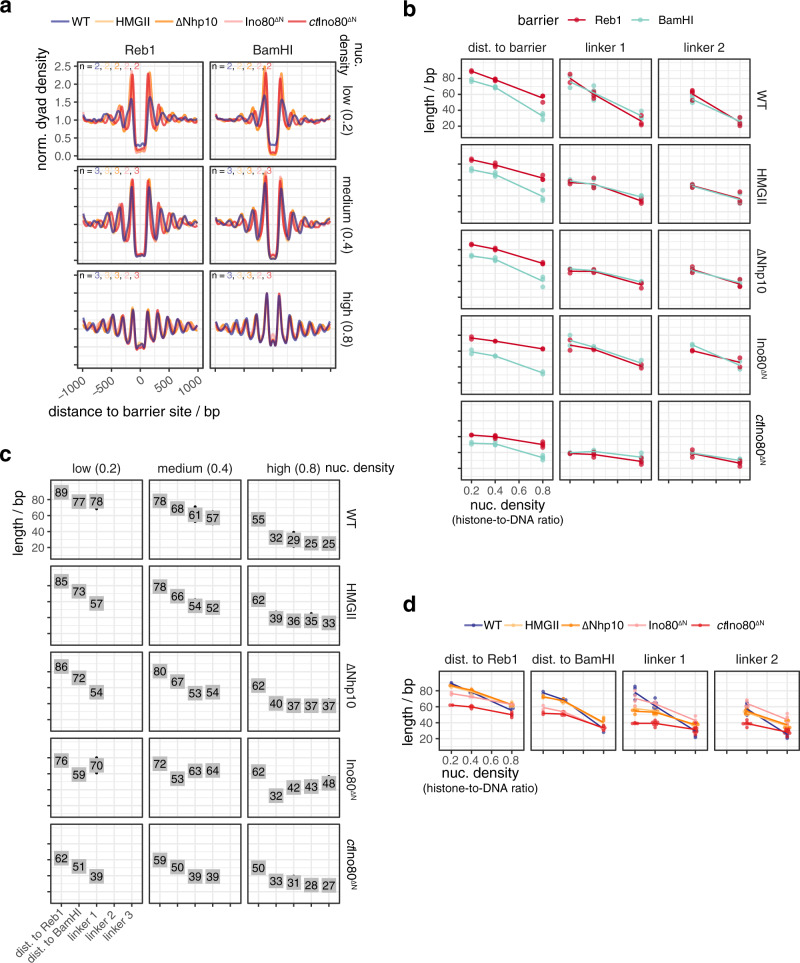
Mutations in the INO80 Nhp10 module affect the generation of array features. **a**–**d** As Fig. 5a–d, respectively, but for the indicated WT and mutant INO80 complexes. Composite plots of individual replicates in Supplementary Fig. 5a. Data for WT INO80 are the same as in Fig. 5.

All mutant complexes were assayed like the wild type (WT) INO80 complex (Figs. 5a–e and 6a–d and Supplementary Figs. 1a and 5a, b). WT INO80 was assayed again alongside with matching SGD chromatin. Comparing these replicates (Fig. 5c) with previous values for WT INO80 (Fig. 2d) reflected variability in preparing SGD chromatin but at the same time the robustness of the overall effects. All tested INO80 mutants generated steady-state patterns (Supplementary Fig. 5b) and differed from WT INO80 in forming aligned arrays in the following ways.

First, all mutants, besides the HQ1/2 mutant, which was almost inactive (Supplementary Fig. 5a), as expected^40^, generated phased regular arrays, but with varying effectiveness and altered distance to one or both barrier types and/or linker lengths compared to WT INO80 (Figs. 5 and 6). This revealed that also INO80 has a ruler, to which both the Arp8 and the Nhp10 module contribute.

Second, the HQ1 showed stronger effects than the HQ2 mutation (Fig. 5d). Both increased the distances to both barriers. While HQ2 increased linker length at all densities, HQ1 gained clamping activity, i.e., linker length hardly depended on nuclesome density. Both mutations uncoupled distance to DNA ends from linker lengths, in contrast to WT INO80 (Fig. 2d, e). Only for HQ1, linker 1 length depended on barrier type (Fig. 5b). We concluded that the Arp8 module, especially via HSAα1 helix-DNA interactions, is threefold involved in spacing, alignment to barrier and responding to nucleosome density.

Third, the Nhp10 module subunits contributed to the ruler mainly through the HMG box of Nhp10 as the respective point mutations (HMGII mutant) mimicked the effects upon lack of all Nhp10 module subunits (ΔNhp10 mutant) (Fig. 6c, d). This was also true with regard to reduced nucleosome binding affinity, which was published for the ΔNhp10 mutant^56^ and also found here for the HMGII mutant (Supplementary Fig. 6). With these mutations, distances to both barriers were not much affected, but linker length depended less on density, i.e., clamping was gained, similar to the HQ1 mutation. Effects of the combined HMGII-HQ1 and -HQ2 mutations were dominated by the HQ mutations, but with reduced effects on distance to barriers (Fig. 5e). Even though the Nhp10 HMG box was a prime candidate for sensing extranucleosomal DNA, its contribution was minor compared to the HSA helix contribution.

Fourth, the INO80^ΔN^ mutation affected the distance to Reb1 and even more to DNA ends, but gained clamping less strongly than the HMGII or ΔNhp10 mutations (Fig. 6d). The INO80^ΔN^ mutant lacked the complete Nhp10 module, but also the Ino80 ATPase N-terminus and Taf14 (Supplementary Fig. 1a), which may account for the differential effects.

Fifth, the INO80^ΔN^, HQ1, and HQ2 mutations most drastically affected distance to BamHI sites, but in opposite ways (Figs. 5c, d and 6c, d).

### *Chaetomium thermophilum* INO80 core complex suggests species-specific ruler

The INO80 core complex of *C. thermophilum*, which we previously used for cryoEM studies^54^, corresponds to the *S. cerevisiae* INO80^ΔN^ mutant as it also lacks its Ino80 ATPase N-terminus. It showed stronger clamping and generated shorter linkers and distances to Reb1, but not to DSBs, than INO80^ΔN^ at all densities, and much shorter linkers and distances to both barriers than *S. cerevisiae* WT INO80 at low and medium densities (Fig. 6b–d). This suggests that INO80’s ruler may be species-specific.

## Discussion

Our study answers one of the oldest questions in chromatin research: what determines the spacing and phasing distances of nucleosome arrays in absolute terms? We find that ATP-dependent remodelers with spacing activity from the ISWI, CHD, and INO80 families do not only equalize linker lengths but bear rulers for setting distances between two adjacent nucleosomes and between nucleosomes and other alignment points.

### Remodeler rulers explain previous in vivo observations

**Fig. 7:**
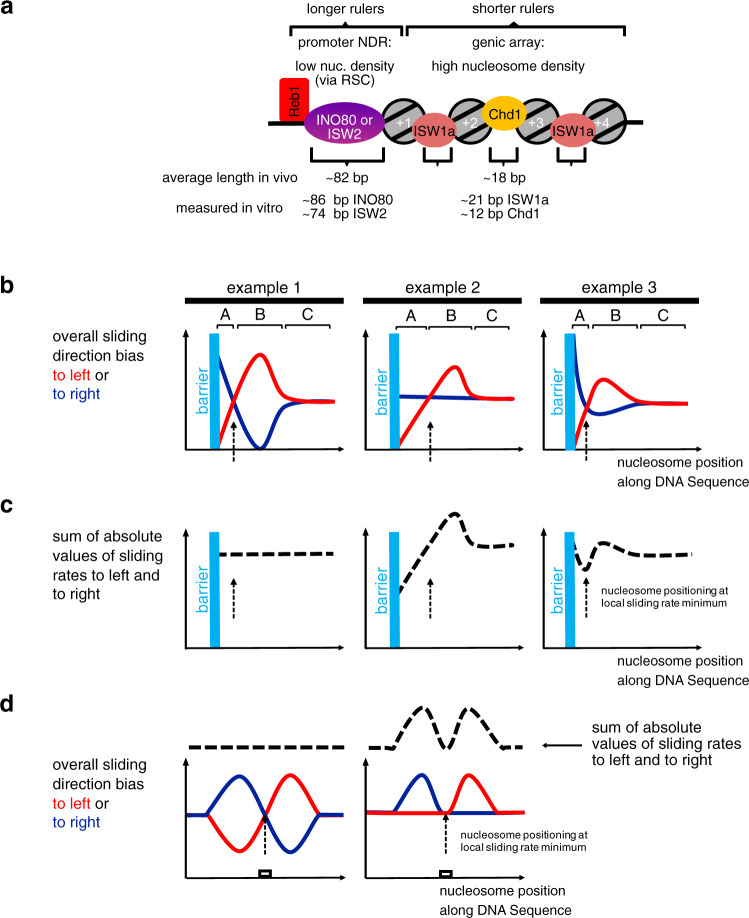
Models for in vivo role of remodeler rulers and for ruler mechanism. **a** Schematic showing how different remodelers contribute to the stereotypical nucleosome organization at 5′ ends of genes in vivo. The promoter is a nucleosome-depleted region (NDR) with low nucleosome density, e.g., due to nucleosome eviction by RSC. The gene body is organized in densely packed and regular nucleosomal arrays. The in vivo observed average distance between the flank of the +1 nucleosome and the center of a Reb1-binding site (distance to barrier) and the average linker length (spacing) mainly reflect the contribution to varying degrees of INO80’s or ISW2’s long ruler or of ISW1a’s or Chd1’s short ruler elements, respectively, as determined in vitro for low or high nucleosome density, respectively, in our study. This explains the main trends, while variations in remodeler type, nucleosome density as well as transcription, replication, and epigenetic marks may further locally modulate nucleosome organization. **b** Three hypothetical examples for how a remodeler ruler regulates the overall bias of sliding a nucleosome to left (red curves) or to right (blue curves) resulting in nucleosome positioning (stippled vertical arrows) in the vicinity of a barrier. **c** As **b**, but plotting sum of absolute values of sliding rates to the left and to the right (stippled black curves). **d** Two hypothetical examples for how a remodeler ruler leads to nucleosome positioning over a DNA sequence element (white box). Symbolics as in **b** and **c**. For details see text.

Rulers combined with barriers mechanistically explain in vivo observations that involved ISW1a, ISW2, Chd1, and INO80 in +1 nucleosome positioning and/or array regularity and phasing^7,13,21–23,26,57–60^ (Fig. 7a).

The average *S. cerevisiae* linker length of 18 bp^61^ results from combined contributions of ISW1a and Chd1^7^. As we show that ISW1a and Chd1 rulers generate linkers of about 21 and 12 bp, respectively, the 18 bp average linker speaks for ISW1a contributing globally more than Chd1. Indeed, lack of Isw1 in vivo globally shortened linkers, while lack of Chd1 affected global spacing only mildly^7,21^. Locally, high transcription rate correlates with shorter spacing^7,9^, which points to increased Chd1 contribution, probably due to increased Chd1 recruitment by elongating RNA polymerase^62^.

Remodeler-specific rulers explain how ISW1a, ISW2, and INO80 affect +1 nucleosome positioning in vivo^21,26,58–60^ and in vitro^18^, especially in combination with RSC. RSC and SWI/SNF are the only yeast remodelers that disassemble nucleosomes^28,29^, particularly at promoter NDRs^12,13,17,21,26,63–68^. By definition, a promoter NDR has lower than average nucleosome density. Therefore, remodeler rulers will set distances to NDR-bound barriers as measured here at low or medium nucleosome density. The in vivo distance between Reb1 and +1 nucleosomes is on average 82 ± 33 bp (median 76 bp; distances between all anti-Reb1-SLIM-ChIP sites at promoters and respective +1 nucleosomes in our in vivo MNase-seq data; with Reb1-PWM sites the mean is very similar but the spread is more narrow (Supplementary Fig. 3b, where downstream distances to Reb1 in groups 1 and 2 also correspond to average of promoter-averaged in vivo distances between Reb1 and +1 nucleosomes from three data sets); see also ref. ^69^ and our accompanying paper^41^ for determination of similar distances by ChIP-exo), which is within in vitro remodeler-specified distances to Reb1 at medium or low density (81–86 bp for INO80, 70–74 bp for ISW2, 58–60 bp for ISW1a). ISW2 and INO80 contribute more to +1 nucleosome positioning in vivo than ISW1a^21^ as their long rulers are more suited for setting long distances across NDRs (Fig. 7a). Setting such long distances contributes to NDR formation, which explains why promoter NDRs of many genes depend on INO80^70^. Conversely, the short Chd1-ruler hardly contributes to +1 positioning and NDRs in vivo^7,13,21^. These different ruler characteristics explain why ISW1a and Chd1 are mainly involved in spacing nucleosomes into densely packed arrays and why ISW2 and INO80 mainly use their ruler for +1 alignment at NDRs in vivo (Fig. 7a). This resolves the conundrum^18^ why yeast has two remodelers, INO80 and ISW2, that seemingly generate “too wide” spacing in vitro compared to average in vivo spacing. Nonetheless, both INO80 and ISW2 may also contribute to in vivo spacing, e.g., at inactive genes^7,71^ or in the absence of other remodelers as shown in a parallel study^72^, and ISW1a may contribute to some +1 nucleosome positions^26,60^ and local fluctuations in nucleosome density, e.g., via RSC (see below) may further modulate nucleosome positioning. We do not preclude that other mechanisms besides ruler features, like recruitment via histone modifications or transcription factors, also affect where each remodeler is active.

### Functional and structural identification of remodeler rulers

The protein ruler model was first proposed for ISW1a^39^. It suggested that ISW1a shortens the linker until its ruler contacts the neighboring nucleosome, but did not conceptualize why this would lead to a stable nucleosome position. We built on and expanded this model, identified remodeler rulers via their functionality, and pinpointed the INO80 ruler also in structural terms. On the functional level, a ruler is revealed if

1. the same remodeler generates the same phasing and/or spacing distances although it works on chromatin with varying nucleosome density (clamping activity), and
2. different remodelers generate different (remodeler-specific) phasing and/or spacing distances although they all work on the same chromatin.

For the INO80 complex the combined criteria (1) and (2) (remodeler-specific clamping) were not fulfilled, but we found by testing INO80 mutant complexes that the Nhp10 module, especially the Ino80 N-terminus, as well as the Arp8 module, especially the Ino80-HSA-helix, contributed to the INO80-generated phasing/spacing distances. This revealed INO80’s ruler as it amounts to a variant of criterion (2) that is sufficient on its own, i.e., different mutant versions of a remodeler generate different phasing/spacing distances although they all work on the same chromatin. Lack of the Ino80 N-terminus, concomitant with lacking the Nhp10 module, allowed INO80, e.g., to slide nucleosomes closer to DNA ends, maybe for steric reasons, while impaired DNA traction during remodeling due to compromised Ino80-HSA helix-DNA interactions had the opposite effect. It remains to be elucidated how exactly such modules within the multi-subunit organization relay barrier information to the core ATPase.

### Remodeler rulers regulate nucleosome sliding direction bias in response to information in the nucleosome environment

We propose an overarching framework for this relay that amounts to a widely applicable remodeler ruler principle (Fig. 7b–d). A remodeler may slide a nucleosome either to the left or to the right from a given position. If there is no bias for sliding in either direction, the nucleosome will experience a random walk along the DNA (regions C in three hypothetical examples in Fig. 7b). Net nucleosome movement in one direction^33–35,43,48,73,74^ requires an overall sliding direction bias in this direction. We conceptualize a remodeler ruler as a remodeler-intrinsic feature that generates an overall sliding direction bias in response to the (epi)genetic information in the environment of the nucleosome that the remodeler is remodeling. The bias may originate from differences, e.g., in binding orientation, ATPase activity, sliding rate or processivity and is regulated by interaction of the ruler with a generalized “barrier”. This may be a GRF, a DSB, a neighboring nucleosome, or a DNA sequence element. Histone modifications/variants may modulate as well. While the microscopic details may differ for different remodelers and information input, the overall regulation of sliding direction bias by the ruler will share three key elements that constitute the ruler mechanism. First, the ruler has a certain reach (regions A + B in Fig. 7b), within which it interacts with the barrier. Second, if the position, from where the remodeler slides the nucleosome, is within region B, the interaction between ruler and barrier biases overall sliding direction toward the barrier (red curve is above blue curve), e.g., due to binding energy gained upon orienting the remodeler toward vs. away from the barrier. Third, if the nucleosome is in region A, the ruler-barrier interaction disfavors sliding toward relative to sliding away from the barrier (blue curve is above red curve), e.g., because the ruler gets sterically in the way. Our study determined the length of region A for different remodeler and barrier types and conditions. Region B and exact curve shapes will have to be determined in future studies. If these three key elements are met, resulting fluxes lead to steady-state nucleosome placement at a defined position relative to the barrier (stippled vertical arrows throughout Fig. 7b–d). This position is a self-stabilizing dynamic equilibrium point (intersection of red and blue curves) without sliding direction bias here, but with biases toward this point from neighboring positions. This model applies to how a remodeler with ruler stably positions a nucleosome next to a GRF as well as to another nucleosome and therefore explains both spacing and phasing.

It also explains density-independent clamping. As long as a remodeler is processive enough to fortuitously bring nucleosomes into region B of a barrier also at low density, the ruler mechanism will keep the nucleosome at the dynamic equilibrium point. Nonetheless, the model can also accommodate sensing of nucleosome density and barrier type, e.g., if the ruler offers a hierarchy of interaction points that depends on density or barrier type. For example, INO80 may be able to adopt different conformations that may provide different interaction sites and have different footprint sizes, which may explain why INO80 can remodel arrays with just 30 bp linkers despite a measured footprint of >50 bp^75^. INO80 mutants showed not concerted but uncoupled effects on distances to Reb1, DNA ends, and nucleosomes, even if the same module, like the Nhp10 module, was differentially mutated. Chd1 generated shorter linker lengths (12–16 bp) than distances to DNA ends or Reb1 (35–40 bp). For Chd1, Reb1 may be a “hard” barrier while nucleosomes are “soft” barriers as they are partially “invaded” by the ruler. Indeed, Chd1 partially unwraps nucleosomal DNA^42^. The way how different remodeler rulers interact with different barriers requires clarification, and we outline our model (Fig. 7b–d) in terms of extension-less point particles, but actual footprints have to be taken into account.

The model is fully compatible with the ruler, i.e., the DBD of Chd1^48^ or *Drosophila* ACF^34^, introducing bias via sensing extranucleosomal DNA length. Indeed, differently long extranucleosomal DNA in mono- or oligonucleosome sliding assays amounts to different distances to barriers like DNA ends or other nucleosomes. Our model is fully consistent with previous data and models, but offers an alternative interpretation and is more widely applicable, e.g., to explain stable nucleosome positioning at only one barrier and not only inbetween two barriers.

We introduced our model in terms of overall sliding direction bias. More specifically, the model may refer to differential regulation of sliding rates, i.e., the *y*-axis in Fig. 7b could correspond to “overall sliding rate to the left or to the right”. If sliding rates are reciprocally regulated (example 1, Fig. 7b), the sum of absolute sliding rate values is constant at each position (Fig. 7c), but not upon asymmetric regulation of sliding direction (examples 2 and 3, Fig. 7b). As special case (example 3, Fig. 7b, c), the dynamic equilibrium point may correspond to a minimum of absolute sliding rate. This case corresponds to the “kinetic release” model^76,77^, which posits that remodelers position nucleosomes at sites where the nucleosome is the (locally) poorest substrate for remodeling.

### Ruler-regulated sliding: the unifying principle for nucleosome positioning by remodelers

As nucleosome positions are defined, but not necessarily determined, by the DNA sequences bound by the histone octamer, all mechanisms that generate consistent nucleosome positions across many DNA molecules of the same sequence, must select certain DNA sequences in competition with other sequences. As shown here and in the accompanying paper^41^, remodelers may mediate this selection in two ways. On the one hand, a remodeler may directly choose a sequence, e.g., INO80 turns DNA shape/mechanics features into +1 nucleosome positions at promoters (accompanying paper^41^). On the other hand, a remodeler ruler may place a nucleosome at a ruler-determined distance to a barrier, e.g., ISW2 aligns nucleosomes to Reb1 and generates a regular array by aligning a second nucleosome to the first and so on. In the former case, the resulting nucleosomal sequence is directly selected for its sequence features, while in the latter case, it is indirectly selected without regards for its sequence features but merely for its position relative to the barrier, as we show here by using Reb1 sites in *S. pombe* and *E. coli* genomes.

Our ruler model unifies these positioning mechanisms. The generalized barrier also encompasses DNA sequence elements, with which a remodeler ruler interacts such that sliding direction bias is regulated (Fig. 7d). This explains observations for hybrid Chd1 remodelers where the Chd1 DBD was replaced with heterologous sequence-specific DBDs^48,78,79^. Such hybrid Chd1 remodelers slide nucleosomes faster toward the cognate site of the heterologous DBD, if it was in reach of this site, until the nucleosome became positioned on the site. In our model, the heterologous DBD is a remodeler ruler. As a DNA sequence element as barrier is no hindrance for nucleosome sliding, the remodeler may slide the nucleosome onto this site. This prevents ruler binding to the site, abolishes the increase in sliding rate linked to ruler binding and makes a nucleosome on the cognate site a poorer nucleosome sliding substrate than at neighboring positions (Fig. 7d, right), which corresponds to the kinetic release model as noted^48^. Our model now adds that sliding from neighboring positions will always (within ruler reach) convene at the cognate site and stabilize this position, even if there is no local sliding rate minimum, as long as the ruler regulates sliding direction bias according to the three key elements outlined above (Fig. 7d, left). As our INO80 mutations differently affected nucleosome positioning via DNA shape/mechanics (accompanying paper^41^) vs. relative to Reb1 vs. DNA ends vs. nucleosomes, the ruler elements seem to be multilayered and maybe linked to different structural conformations. For example, the INO80 conformation required for aligning nucleosomes at high density may not be compatible with positioning +1 nucleosomes via DNA shape/mechanics. Nonetheless, as the Ino80-HSA mutations affected nucleosome positioning both via DNA shape/mechanics (accompanying paper^41^) and relative to barriers (this study), the Ino80-HSA domain is a functionally crucial part of the INO80 ruler.

In vivo there are many ways that may regulate nucleosome positioning by remodelers, e.g., by recruitment, by architectural factors, by nucleosome density fluctuations, or by histone variants and modifications, possibly in the context of elongating polymerases. Nonetheless, we expect that the regulation of nucleosome sliding direction bias via built-in sensing and processing of information in the nucleosome environment, i.e., a remodeler ruler, will be at the heart of each nucleosome positioning mechanism.

## Methods

### Embryonic *D. melanogaster* histones, whole-genome plasmid libraries, and salt gradient dialysis

#### Embryonic *D. melanogaster* histone purification

The preparation of embryonic *D. melanogaster* histone octamers was carried out as described^80,81^. Briefly, 50 g of 0–12 h old *D. melanogaster* embryos (strain OregonR) were dechorionated in 3% sodium hypochlorite, washed with dH_2_O, and resuspended in 40 ml lysis buffer (15 mM K·HEPES pH 7.5, 10 mM KCl, 5 mM MgCl_2_, 0.1 mM EDTA, 0.5 mM EGTA, 1 mM DTT, 0.2 mM PMSF, 10% glycerol). Embryos were homogenized (Yamamoto homogenizer), filtered through cloth, and centrifuged at 6500 × *g* for 15 min. Nuclei (brownish light pellet) were washed three times with 50 ml sucrose buffer (15 mM K·HEPES pH 7.5, 10 mM KCl, 5 mM MgCl_2_, 0.05 mM EDTA, 0.25 mM EGTA, 1 mM DTT, 0.2 mM PMSF, 1.2% sucrose) and resuspended in 30 ml sucrose buffer containing 3 mM CaCl_2_. To obtain mononucleosomes, nuclei were incubated for 10 min at 26 °C with 6250 Units MNase (Sigma-Aldrich). Reaction was stopped with 10 mM EDTA, nuclei were pelleted and resuspended in 6 ml TE (10 mM Tris·HCl pH 7.6, 1 mM EDTA) containing 1 mM DTT and 0.2 mM PMSF followed by 30–45 min of rotation at 4 °C. Nuclei were centrifuged for 30 min at 15,300 × *g* at 4 °C. Solubilized mononucleosomes in the supernatant were applied to a hydroxyapatite column pre-equilibrated with 0.63 M KCl, 0.1 M potassium phosphate pH 7.2, 1 mM DTT. After washing the hydroxyapatite column with 0.63 M KCl, 0.1 M potassium phosphate pH 7.2, 1 mM DTT, histone octamers were eluted with 2 M KCl, 0.1 M potassium phosphate pH 7.2, 1 mM DTT, concentrated and stored at −20 °C after adjusting to 50% glycerol and 1x Complete (Roche) protease inhibitors without EDTA.

#### Whole-genome plasmid library expansion

The clone collection of the *S. cerevisiae* genomic plasmid library (pGP546^82^; Open Biosystems) was expanded (8–12 rounds, three replicas) with a Singer ROTOR plating machine (Singer Instruments). Plates were incubated for 16 h at 37 °C. Colonies from all plates were combined in 3 × 2 l of LB medium plus 50 µg/ml kanamycin and grown for 4 h at 37 °C with shaking. Plasmids were prepared via Plasmid Giga Preparation (PC 10,000 Kit, Macherey & Nagel).

For *S. pombe* and *E. coli* plasmid library generation, genomic *S. pombe* (Hu303) and *E. coli* (B strain, ATCC 11303 strain, 14380, Affymetrix) DNA was fragmented by a limited SauIIIA or AluI digest, ligated into pJET1.2 vector (Thermo Fisher Scientific), and transformed into electrocompetent DH5α cells. Cells were plated on LB medium plus 100 µg/ml ampicillin, grown for 16–20 h at 37 °C, combined in LB medium plus 100 µg/ml ampicillin and grown for another 4 h. Plasmids were extracted with Plasmid Mega Preparation Kit (PC 2000 Kit, Macherey & Nagel).

#### Salt gradient dialysis (SGD)

For low, medium, and high assembly degrees, 10 µg of plasmid library DNA (*S. cerevisiae*, *S. pombe* or *E. coli*) were mixed with ~2, 4, or 8 µg of *Drosophila* embryo histone octamers, respectively, in 100 µl assembly buffer (10 mM Tris·HCl pH 7.6, 2 M NaCl, 1 mM EDTA, 0.05% IGEPAL CA630 (Sigma), 0.2 µg/µl BSA). Samples were transferred to Slide-A-lyzer mini dialysis devices (MWCO 3.5 kDa; Thermo Fisher Scientific, cat no. 69550), which were placed in a 3 l beaker containing 300 ml of high salt buffer (10 mM Tris·HCl pH 7.6, 2 M NaCl, 1 mM EDTA, 0.05% IGEPAL CA630, 14.3 mM β-mercaptoethanol), and dialyzed against a total of 3 l low salt buffer (10 mM Tris·HCl pH 7.6, 50 mM NaCl, 1 mM EDTA, 0.05% IGEPAL CA630, 1.4 mM β-mercaptoethanol) added continuously while stirring via a peristaltic pump over a time course of 16 h. β-mercaptoethanol was always added freshly. After complete transfer of low salt buffer, samples were dialyzed against 1 l low salt buffer for 1 h at room temperature. DNA concentration of the SGD chromatin preparations was estimated with a DS-11+ spektrophotometer (Denovix). SGD chromatin could be stored at 4 °C for several weeks. To estimate the extent of the assembly degree, an aliquot of the sample was subjected to MNase digestion (below) for MNase-ladder read out.

### Purifications of chromatin remodeling enzymes

#### Expression and purification of WT and mutant INO80 complex

Exact strategy for recombinant expression of *S. cerevisiae* INO80 complex in insect cells and complex purification is described in the accompanying paper^41^. Briefly, MultiBac technology^83^ was applied to generate two baculoviruses carrying coding sequences for *S. cerevisiae* Ino80 (2xFlag), Rvb1, Rvb2, Arp4, Arp5-His, Arp8, Actin, Taf14, Ies1, Ies2, Ies3, Ies4, Ies5, Ies6, and Nhp10 (HMGII mutant using respective primer sequences in Supplementary Table 1), which were subcloned into pFBDM vectors and sequence verified by Sanger Sequencing. High Five (Hi5) insect cells (BTI-TN-5B1-4 Invitrogen) were co-infected with two or three baculoviruses 1/100 (v/v) each for expression purposes. The recombinantly expressed WT or mutant INO80 complexes were purified from insect cells as described^56^, which resulted in a pure and monodisperse sample. Shortly, cells were resuspended in lysis buffer (50 mM Tris·HCl pH 7.9, 500 mM NaCl, 10% glycerol, 1 mM DTT, SIGMAFASTTM protease inhibitor cocktail), sonified (Branson Sonifier, 3× 20 s with 40% duty cycle and output control 3–4) and cleared by centrifugation (Sorvall Evolution RC, SS34 rotor, 15,000 × *g*). The supernatant was incubated with anti-Flag M2 Affinity Gel (Sigma-Aldrich) and centrifuged for 15 min at 1000 × *g* and 4 °C. The anti-Flag resin was washed with buffer A (25 mM K-HEPES pH 8.0, 500 mM KCl, 10% glycerol, 0.025 mM IGEPAL CA630, 4 mM MgCl_2_, 1 mM DTT) and buffer B (25 mM K-HEPES pH 8.0, 200 mM KCl, 10% glycerol, 0.02 mM IGEPAL CA630, 4 mM MgCl_2_, 1 mM DTT). Recombinant INO80 complex was eluted with buffer B containing 0.22 mg/ml Flag Peptide (Sigma-Aldrich). Anion exchange chromatography (MonoQ 5/50 GL, GE Healthcare) was used for further purification which resulted in a monodisperse and clear INO80 complex. Using standard cloning techniques, three INO80 (2xFlag) HSA domain mutants (HQ1, HQ2, HQ1/2, Fig. 4d), one N-terminal deletion mutant (Ino80^ΔN^, deletion of the first 461 amino acids of the N-terminus of Ino80) and two INO80 (2xFlag) Nhp10 module mutants (ΔNhp10 (INO80 complex without Ies1, Ies3, Ies5, and Nhp10 but with Ino80 N-terminus) and HMGII (Fig. 4g and Supplementary Table 1) were generated and integrated into baculoviruses using MultiBac Technology. Expression and purification of mutant INO80 complexes was carried out as described above. The INO80 core complex from *Chaetomium thermophilum* (equivalent to the *S. cerevisiae* N-terminal deletion mutant) was essentially purified as described^54^.

#### Expression and purification of full-length Chd1 and FACT

Hi5 cells (600 ml) were grown in ESF-921 media (Expression Systems) and infected with V1 virus for full-length Chd1 (tagged with a N-terminal 6 × His tag, followed by a MBP tag, and a tobacco etch virus protease cleavage site) or FACT (Spt16 carries an N-terminal 6 × His tag, followed by an MBP tag, and a tobacco etch virus protease cleavage site) for protein expression. Cells were grown for 72 h at 27 °C and subsequently harvested by centrifugation (238 × *g*, 4 °C, 30 min). Supernatant was discarded and cell pellets resuspended in lysis buffer (300 mM NaCl, 20 mM Na·HEPES pH 7.4, 10% (v/v) glycerol, 1 mM DTT, 30 mM imidazole pH 8.0, 0.284 μg/ml leupeptin, 1.37 μg/ml pepstatin A, 0.17 mg/ml PMSF, 0.33 mg/ml benzamidine). Resuspended cells were snap frozen and stored at −80 °C.

All protein purifications were performed at 4 °C. Frozen cell pellets were thawed and lysed by sonication. Lysates were cleared using centrifugation (18,000 × *g*, 4 °C, 30 min and 235,000 × *g*, 4 °C, 60 min). The supernatant containing Chd1 was filtered with 0.8-μm syringe filters (Millipore) and applied onto a GE HisTrap HP 5 ml (GE Healthcare). The column was washed with ten column volumes (CV) lysis buffer, 5 CV lysis buffer with high salt (1 M NaCl, 20 mM Na·HEPES pH 7.4, 10% (v/v) glycerol, 1 mM DTT, 30 mM imidazole pH 8.0, 0.284 μg/ml leupeptin, 1.37 μg/ml pepstatin A, 0.17 mg/ml PMSF, 0.33 mg/ml benzamidine), and 5 CV lysis buffer. Chd1 was eluted using a 40-min gradient (flow rate 1.5 ml/min) of 0–100% elution buffer (300 mM NaCl, 20 mM Na·HEPES pH 7.4, 10% (v/v) glycerol, 1 mM DTT, 500 mM imidazole pH 8.0, 0.284 μg/ml leupeptin, 1.37 μg/ml pepstatin A, 0.17 mg/ml PMSF, 0.33 mg/ml benzamidine). Fractions containing Chd1 were pooled and subjected to dialysis/TEV protease digestion for 16 h (300 mM NaCl, 20 mM Na·HEPES pH 7.4, 10% (v/v) glycerol, 1 mM DTT, 30 mM imidazole with 2 mg His6-TEV protease).

The dialyzed sample was again applied to a GE HisTrap HP 5 ml. The flow-through, which contained cleaved tag-less Chd1, was concentrated using an Amicon Millipore 15 ml 50,000 MWCO centrifugal concentrator. The concentrate was applied to a GE S200 16/600 pg size exclusion column in 300 mM NaCl, 20 mM Na·HEPES pH 7.4, 10% (v/v) glycerol, 1 mM DTT. Fractions containing Chd1 were concentrated to ~100 μM. The sample was aliquoted, snap frozen, and stored at −80 °C.

FACT was purified as above with minor modifications. After dialysis, the sample was subjected to a tandem GE HisTrap HP 5 ml and GE HiTrap Q 5 ml columns combination. After sample application, the columns were washed with lysis buffer and the HisTrap removed. FACT was eluted by applying a high salt buffer gradient from 0 to 100% high salt buffer (1 M NaCl, 20 mM Na·HEPES pH 7.4, 10% (v/v) glycerol, 1 mM DTT, 30 mM imidazole pH 8.0). Fractions with FACT were applied to a GE S200 16/600 pg size exclusion column. Peak fractions with FACT were concentrated to a concentration of ~60 µM, aliquoted, snap frozen, and stored at −80 °C.

#### Expression and purifications of ISW1a, ISW2, and Fun30

Tandem affinity purification of ISW1a (TAP-Ioc3) and Fun30 (TAP-Fun30) was performed as follows: cultures were grown in YPD media, harvested cells were washed once with water. The cells were lysed in buffer E (20 mM Na·HEPES pH 7.5, 350 mM NaCl, 10% glycerol, 0.1% Tween, and 0.5 mM DTT) and protease inhibitors by grinding in the presence of liquid nitrogen. Lysates were clarified at 40,000 × *g* at 4 °C for 1 h. Cleared lysates were incubated with IgG-Sepharose (GE Healthcare) at 4 °C for 2 h and eluted by TEV protease (Invitrogen) cleavage at 4 °C overnight. The elutions were incubated with calmodulin affinity resin (Agilent Technology) in buffer E plus 2 mM CaCl_2_ at 4 °C for 2 h and eluted in buffer E plus 10 mM EGTA.

ISW2 (2xFLAG-Isw2, YTT480^32^) was purified as follows: cleared lysate was incubated with Anti-FLAG M2 affinity gel (Sigma-Aldrich) at 4 °C for 1 h and eluted with 0.1 mg/ml 3X FLAG peptide (Sigma-Aldrich). E-buffer (20 mM Na·HEPES pH 7.5, 350 mM NaCl, 10% glycerol, 0.1% Tween, and 0.5 mM DTT) was used during the entire purification.

Purified proteins were concentrated with VIVASPIN concentrators (Sartorius) and dialyzed against E-Buffer with 1 mM DTT. Subunit compositions were confirmed by SDS-PAGE (Supplementary Fig. 1a) and mass spectrometry. For full scan images of all gels presented in this study see Source Data.

### Expression and purification of *S. cerevisiae* Reb1

Expression and purification of *S. cerevisiae* Reb1 was exactly as described, i.e., the protein was from the same preparation as in ref. ^18^.

### Genome-wide remodeling reaction

All remodeling reactions, except Chd1-containing reactions, were performed at 30 °C in 100 µl with final buffer conditions of 26.6 mM Na·HEPES pH 7.5, 1 mM Tris·HCl pH 7.6, 85.5 mM NaCl, 8 mM KCl, 10 mM ammonium sulfate, 10 mM creatine phosphate (Sigma-Aldrich), 3 mM MgCl_2_, 2.5 mM ATP, 0.1 mM EDTA, 0.6 mM EGTA, 1 mM DTT, 14% glycerol, 20 ng/µl creatine kinase (Roche Applied Science). Chd1-containing reactions were performed in 100 µl in 26.6 mM Na·HEPES pH 7.5, 1 mM Tris·HCl pH 7.6, 50 mM NaCl, 10 mM creatine phosphate (Sigma-Aldrich), 3 mM MgCl_2_, 2.5 mM ATP, 0.1 mM EDTA, 0.6 mM EGTA, 1 mM DTT, 14 % glycerol, 20 ng/µl creatine kinase.

If called for, 10 nM of remodeling enzyme (but 50 nM Chd1/FACT), 40 nM Reb1, and 20 units of BamHI (NEB) were added. Note that the restriction enzyme was added during the remodeling reaction in contrast to the accompanying study^41^. Before full-length Chd1 (in lysis buffer) was added to the reaction, it was diluted together with FACT from 300 mM NaCl (lysis buffer) to 30 mM NaCl. For that, full-length Chd1 and purified FACT was mixed in a 1.2:1 molar ratio in lysis buffer (300 mM NaCl, 20 mM Na·HEPES pH 7.4, 10% (v/v) glycerol, 1 mM DTT), incubated on ice for 5 min and then diluted to 30 mM NaCl final concentration.

Remodeling reactions were started by adding 10 µl SGD chromatin corresponding to about 1 µg DNA assembled into nucleosomes and terminated after 2 h incubation at 30 °C by adding 0.8 units apyrase (NEB) followed by incubation at 30 °C for 30 min.

Independent replicates of remodeling reactions refer to independent SGD chromatin preparations. The experimental conditions for each sample are detailed in Supplementary Data 1.

### MNase-seq

After apyrase addition, remodeling reactions were supplemented with CaCl_2_ to a final concentration of 1.5 mM and digested with 100 units MNase (Sigma-Aldrich, N5386) to generate mostly monoucleosomal DNA. Chd1-containing reactions were incubated with 20 units MNase to get the same extent of mononucleosomal DNA. In total, 10 mM EDTA and 0.5% SDS (final concentrations) were added to stop the MNase digest. After proteinase K treatment for 30 min at 37 °C, samples were ethanol precipitated and electrophoresed for 1.5–2 h at 100 V in a 1.5% agarose gel with 1x Tris-acetate-EDTA (TAE) buffer. Mononucleosome bands were excised and purified with PureLink Quick Gel Extraction Kit (Thermo Fisher Scientific).

For library preparation, 10–50 ng of mononucleosomal DNA were incubated with 1.25 units Taq polymerase (NEB), 3.75 units T4 DNA polymerase (NEB), and 12.5 units T4-PNK (NEB) in 1x ligation buffer (B0202S, NEB) for 15 min at 12 °C, 15 min at 37 °C, and 20 min at 72 °C. To ligate NEBNext Adaptors (0.75 µM final concentration, NEBNext Multiplex Oligos Kit) to the DNA, samples were incubated with T4 DNA ligase (NEB) at 25 °C for 15 min, followed by incubation with 2 units USER enzyme (NEB) for 10 min at 37 °C. Fragments were purified using two volumes AMPure XP beads (Beckman Coulter) and amplified for 8–10 cycles using NEBNext Multiplex Oligos, Phusion High-Fidelity DNA Polymerase (1 U, NEB), deoxynucleotide solution mix (dNTP, 2.5 mM, NEB) and Phusion HF Buffer (1x, NEB). The following protocol was applied for amplification: 98 °C for 30 s, 98 °C for 10 s, 65 °C for 30 s, 72 °C for 30 s with a final amplification step at 72 °C for 5 min. DNA content was assessed by using Qubit dsDNA HS Assay Kit (Invitrogen). PCR reactions were applied to an 1.5% agarose gel, relevant fragment length (~270 bp) was excised and purified via PureLink Quick Gel Extraction Kit (Thermo Fisher Scientific). DNA was measured again with Qubit dsDNA HS Assay Kit and diluted to a final concentration of 10 nM (calculation based on the assumption that the DNA fragment length is 272 bp, i. e., 147 bp nucleosomal DNA and 122 bp sequencing adapter). Diluted samples were pooled according to sequencing reads (~6 Mio reads/sample). The final pool was quantified with BioAnalyzer (Agilent) and analyzed on an Illumina HiSeq 1500 in 50 bp single-end mode (Laboratory for Functional Genome Analysis, LAFUGA, LMU Munich).

For MNase-seq of in vivo chromatin, nuclei were prepared from strain BY4741 grown to log phase at 30 °C as described^84^. Briefly, cell walls were degraded by Zymolyase (ICN Biochemicals zymolyase 100T) in the presence of 1 M sorbitol and cells lysed in hypoosmotic buffer (18% Ficoll (Sigma-Aldrich, Ficoll Type 400 F4375), 20 mM KH_2_PO_4_, 1 mM MgCl_2_, 0.25 mM EGTA, 0.25 mM EDTA) followed by centrifugation at 22,550 × g for 30 min to collect chromatin (“nuclei”). Nuclei aliquots were digested with MNase in MNase buffer (15 mM Tris-HCl pH 7.5, 50 mM NaCl, 1.4 mM CaCl_2_, 0.2 mM EGTA, 0.2 mM EDTA, 5 mM 2-mercaptoethanol) to yield mostly mononucleosomal DNA fragments. Digestion was stopped by SDS and EDTA and DNA purified by digestion with proteinase K, phenol extraction, ethanol precipitation, RNaseA digestion, and isopropanol precipitation. DNA was resolved in an 1.5% agarose gel in 1x TAE buffer and mononucleosomal fragments were excised, purified, and used for sequencing library preparation as above.

### Data processing

Sequencing data were mapped to the *S. cerevisiae* (SacCer3, R64-1-1), *S. pombe* (EF2), or *E. coli* strain B (REL606, Assembly GCA_000017985.1) genome (see also “Data availability”) using Bowtie^85^. Multiple matches were omitted. After mapping, data were imported into R Studio using GenomicAlignments^86^. Every read was shifted by 73 bp to cover the nucleosome dyad and extended to 50 bp. Genome coverage was calculated and either aligned to in vivo +1 nucleosome positions (annotated according to TSS^87^ and our own in vivo MNase-seq data, see accompanying paper^41^, and GEO deposition), BamHI cut sites, anti-Reb1 SLIM-ChIP hits^49^, or Reb1 PWM hits^67^ (only for Supplementary Fig. 3a, b). Signal was normalized per gene in a 2001 bp window centered on the alignment point.

Heat maps were sorted either by NFR length (distance between in vivo +1 and −1 nucleosome, annotated by calling nucleosomes of in vivo MNase-seq; see accompanying paper;^41^ see GEO deposition for +1 and −1 nucleosome positions and NFR lengths) or by anti-Reb1 SLIM-ChIP score. For the latter, anti-Reb1 SLIM-ChIP data (GSM2916407) were aligned to in vivo +1 nucleosome positions and sorted by signal strength in a 120 bp window 160 bp upstream of every +1 nucleosome. Cut off for Reb1-bound genes (red shading in Fig. 1c) was top 12.5%.

For calculation of linker length and distance to barrier, peak positions in composite plots either aligned to Reb1 or BamHI (e.g., Fig. 2a, b) were determined by calling the peak maxima in R.

For promotor grouping according to Reb1 site orientation, anti-Reb1 SLIM-ChIP hits, which contain a PWM site (±50 bp) and which are located within 400 bp upstream of in vivo +1 nucleosomes were used. Group 1 contains promotors where the Reb1 PWM motif is located on the sense strand and group 2, where the Reb1 PWM motif is located on the antisense strand. Group 3 contains Reb1 sites at bidirectional promotors.

### Gel-shift assays

Gel-shift assays were done as described in Knoll et al.^40^. INO80 complex was titrated to constant concentration of 20 nM 0N80 mononucloeosomes and incubated in 25 mM HEPES-KOH pH 8.0, 60 mM KCl, 7% glycerol, and 1 mM CaCl_2_ for 30 min on ice. INO80-bound as well as unbound mononucleosomes were resolved by native PAGE (Novex 4–16% Bis-Tris Protein Gels; Invitrogen) and visualized on a Typhoon FLA 9000 plate reader (GE Healthcare) with 25 μm pixel size, using FITC fluorescence scan. Scanned images were quantified in ImageJ. For visual representation the image was enhanced using linear level adjustment in Affinity Photo.

### Reporting summary

Further information on research design is available in the Nature Research Reporting Summary linked to this article.

## Supplementary information

Supplementary Information

Description of Additional Supplementary Files

Supplementary Data 1

Peer Review File

Reporting Summary

## Data Availability

The data that support this study are available from the corresponding author upon reasonable request. Data generated in the course of this study have been deposited in the NCBI Gene Expression Omnibus with the accession number GSE140614. Previously published Reb1 SLIM-ChIP data are available with the accession number GSM2916407. *S. cerevisiae* transcriptome data used for annotation of TSS is available from ArrayExpress [http://www.ebi.ac.uk/arrayexpress] under accession number E-TABM-590. *S. cerevisiae* (SacCer3, R64-1-1) and *S. pombe* (EF2) genome sequences were retrieved from iGenomes [https://support.illumina.com/sequencing/sequencing_software/igenome.html]. The *E. coli* strain B REL606 genome sequence was retrieved from the NCBI assembly database [https://www.ncbi.nlm.nih.gov/assembly/GCF_000017985.1/]. Source data are provided with this paper.

## Source data

Source Data
