# Supplementary material for: Ruler elements in chromatin remodelers set nucleosome array spacing and phasing
Source: Nat Commun. 2021 May 28;12:3232. doi: 10.1038/s41467-021-23015-0 (PMC8163753; doi:10.1038/s41467-021-23015-0)
Supplement: Supplementary file 1 — Supplementary Information [file 41467_2021_23015_MOESM1_ESM.pdf]

Supplementary Information

Ruler elements in chromatin remodelers set nucleosome array spacing and phasing

Elisa Oberbeckmann[1,2,#], Vanessa Niebauer[3,4,#], Shinya Watanabe[5], Lucas Farnung[6], Manuela Moldt[3,4], Andrea Schmid[1], Patrick Cramer[6], Craig L. Peterson[5], Sebastian Eustermann[3,4,7]*, Karl-Peter Hopfner[3,4]*, Philipp Korber[1]*

[1] Division of Molecular Biology, Biomedical Center (BMC), Faculty of Medicine, LMU Munich, Martinsried near Munich, Germany

[2] current address: Department of Molecular Biology, Max Planck Institute for Biophysical Chemistry, Göttingen, Germany

[3] Gene Center, Faculty of Chemistry and Pharmacy, Ludwig-Maximilians-Universität München, Munich, Germany

[4] Department of Biochemistry, Faculty of Chemistry and Pharmacy, Ludwig-Maximilians-Universität München, Munich, Germany

[5] Program of Molecular Medicine, University of Massachusetts, Worcester, USA

[6] Department of Molecular Biology, Max Planck Institute for Biophysical Chemistry, Göttingen, Germany

[7] European Molecular Biology Laboratory (EMBL), Structural and Computational Biology Unit, Heidelberg, Germany

[#] These authors contributed equally.

* Corresponding authors: pkorber@lmu.de, hopfner@genzentrum.lmu.de, sebastian.eustermann@embl.de

Table of Contents

Supplementary Figures 2

Supplementary Table 1 8

Supplementary References 9

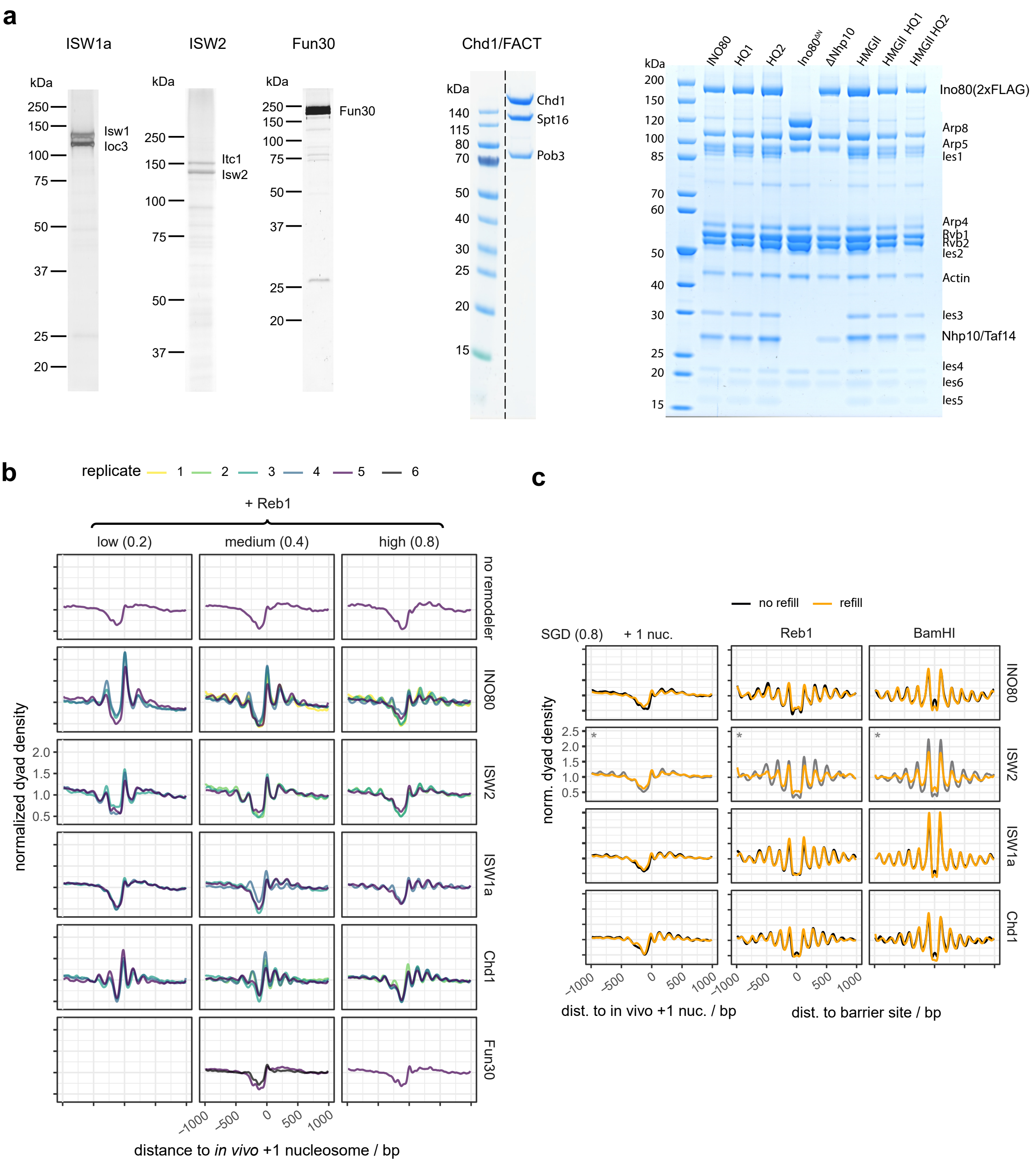

Supplementary Figure 1. **Purified yeast remodelers and plots of individual replicates for samples with INO80, ISW2, ISW1a, Chd1 or Fun30. a** SDS-PAGE analyses of purified remodeler complexes. SDS-PAGE analysis was repeated for every protein purification. Results were similar. Lanes separated by dashed line were electrophoresed on the same gel but not next to each other. **b** Composite plots as in Fig. 1d for individual replicates and the indicated combinations of remodeler, Reb1 and nucleosome density. “no remodeler” denotes absence of remodeler. **c** Composite plots aligned at in vivo +1 nucleosome positions (left), Reb1 (middle,[1]) or BamHI (right) sites for MNase-seq analysis of SGD chromatin assembled at high nucleosome density (SGD (0.8), single replicate) and incubated with the indicated remodelers as in Fig. 1d (no refill) or with doubled remodeler concentration for the second half of incubation time (refill).

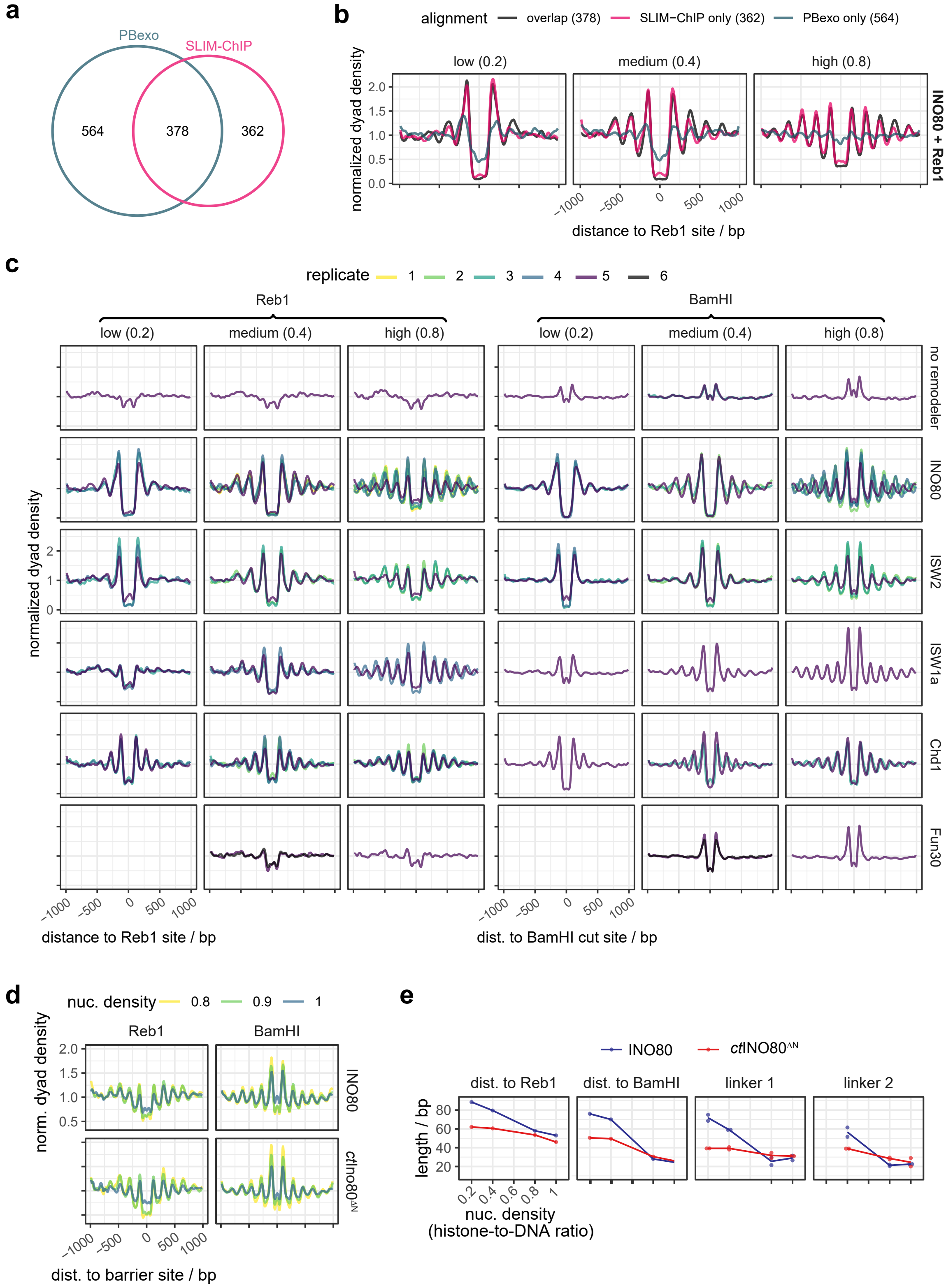

Supplementary Figure 2. **Comparison of Reb1 site annotations and plots of individual replicates for samples with INO80, ISW2, ISW1a, Chd1 or Fun30. a** Venn diagram comparing number of Reb1 binding sites called by only PB-exo[2], only SLIM-ChIP[1] or both. **b** As Fig. 2a but plotted separately for three nucleosome densities (low (0.2), medium (0.4), high (0.8)) and aligned at the Reb1 sites of the three groups (overlap, SLIM-ChIP only, PB-exo only) according to the Venn diagram in (a). **c** As Fig. 2a but for individual replicates and indicated combinations of barrier, remodeler and nucleosome density. “no remodeler” denotes absence of remodeler. **d** As (c), but for the indicated nucleosome densities and *S. cerevisiae* WT INO80 complex versus *C. thermophilum* core INO80 complex (ctINO80$^{\Delta N}$). **e** As Fig. 2e, but for the indicated remodelers (as in (d)) and nucleosome densities.

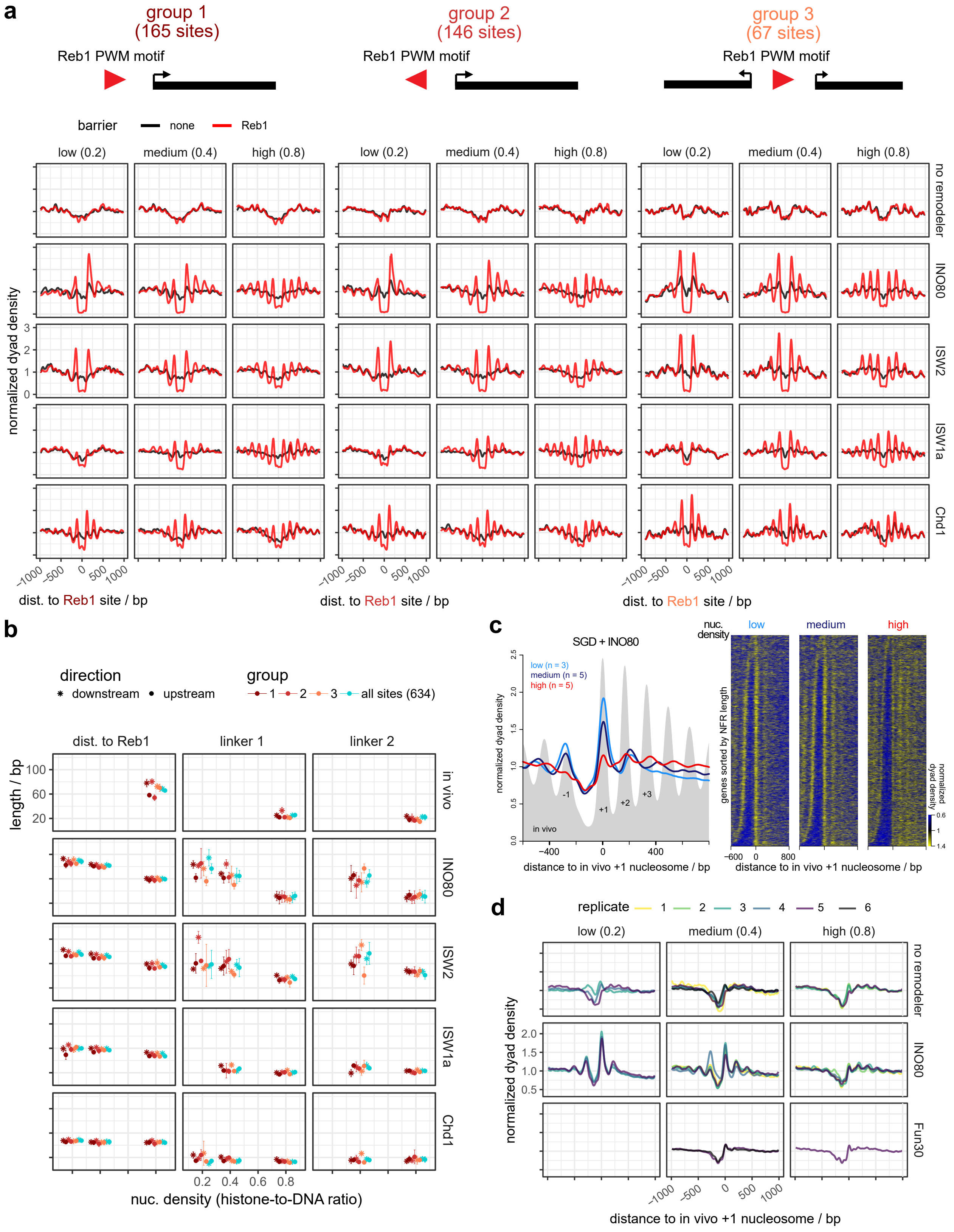

Supplementary Figure 3. **Effects of promoter Reb1 site orientation relative to genes on nucleosome positioning by INO80, ISW2, ISW1a or Chd1 with Reb1, and nucleosome density effects on nucleosome positioning by INO80 and Fun30 on their own. a** As Fig. 2a but aligned at Reb1-PWM positions and for indicated combinations of remodeler and nucleosome density. Anti-Reb1 SLIM-ChIP sites that also contained Reb1 PWM were sorted into groups 1 to 3 (number of sites per group indicated) according to Reb1 PWM orientation relative to one or two genes. Tracks show merged data from replicates in Supplementary Figs. 1b, 2c, 3d and Supplementary data 1; replicate numbers as in Fig. 1d. "no remodeler": absence of remodeler; "none": absence of Reb1. **b** As Fig. 2c, but averages and standard deviation (SD for n ≥ 3) of up- and downstream lengths for groups as in (a) and for all, including outside of promoters, anti-Reb1 SLIM-ChIP sites that contain a Reb1-PWM (light blue). Distances to Reb1 refer to Reb1-PWM positions. Replicate numbers as in Fig. 1d (n = 2 for ISW1a at 0.8; n = 3 for INO80 at 0.2, ISW2 at all, ISW1a at 0.2/0.4, Chd1 at 0.2/0.8; n = 4 for Chd1 at 0.4; n = 5 for INO80 at 0.4/0.8 nuc. densities). Replicates shown in Supplementary Fig. 2c. In vivo MNase-seq data (n = 3) from this study and two others (GSM4193057[3], SRR124142[4]). **c** Composite plots (left) and heat maps (right) of MNase-seq analysis of in vivo chromatin or SGD chromatin reconstituted with the indicated nucleosome density and incubated with recombinant WT INO80. Data are aligned at in vivo +1 nucleosome positions and heat maps are sorted from top to bottom by increasing NDR length. Traces with indicated replicate number (n) represent merged data. Positions of -1, +1, +2, +3 nucleosomes of the in vivo pattern are labeled. **d** Composite plots as in (c), left, but for individual replicates and the indicated remodelers. "no remodeler": absence of remodeler.

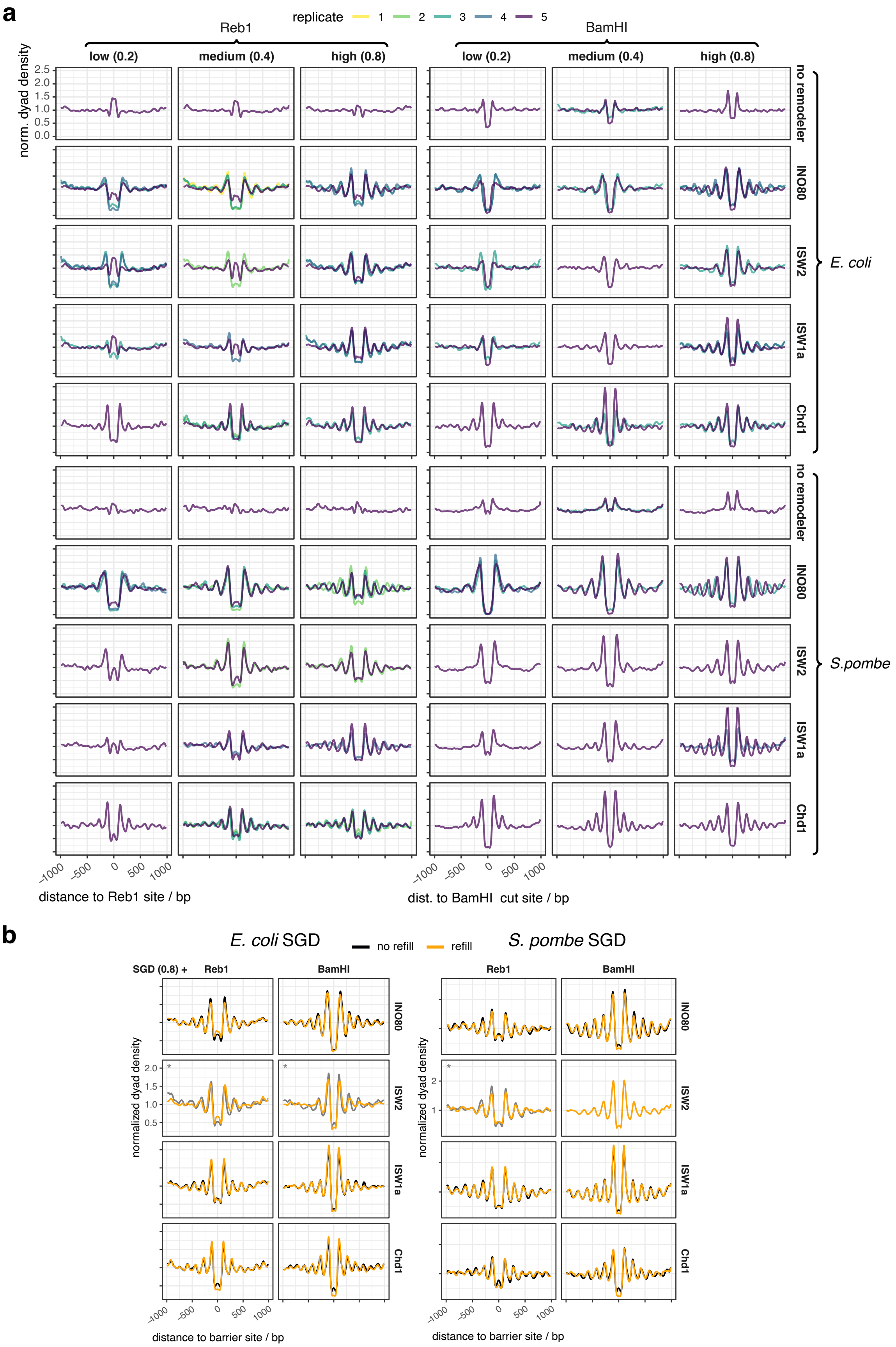

Supplementary Figure 4. **Plots of individual replicates for samples with INO80, ISW2, ISW1a or Chd1 and *E. coli* or *S. pombe* DNA. a** As Supplementary Fig. 2c, but for the indicated genomes. Replicate 5 corresponds to the reconstitution with mixed genomes. **b** As Supplementary Fig. 1c, but for the indicated genomes and remodelers and without alignment at in vivo +1 nucleosomes.

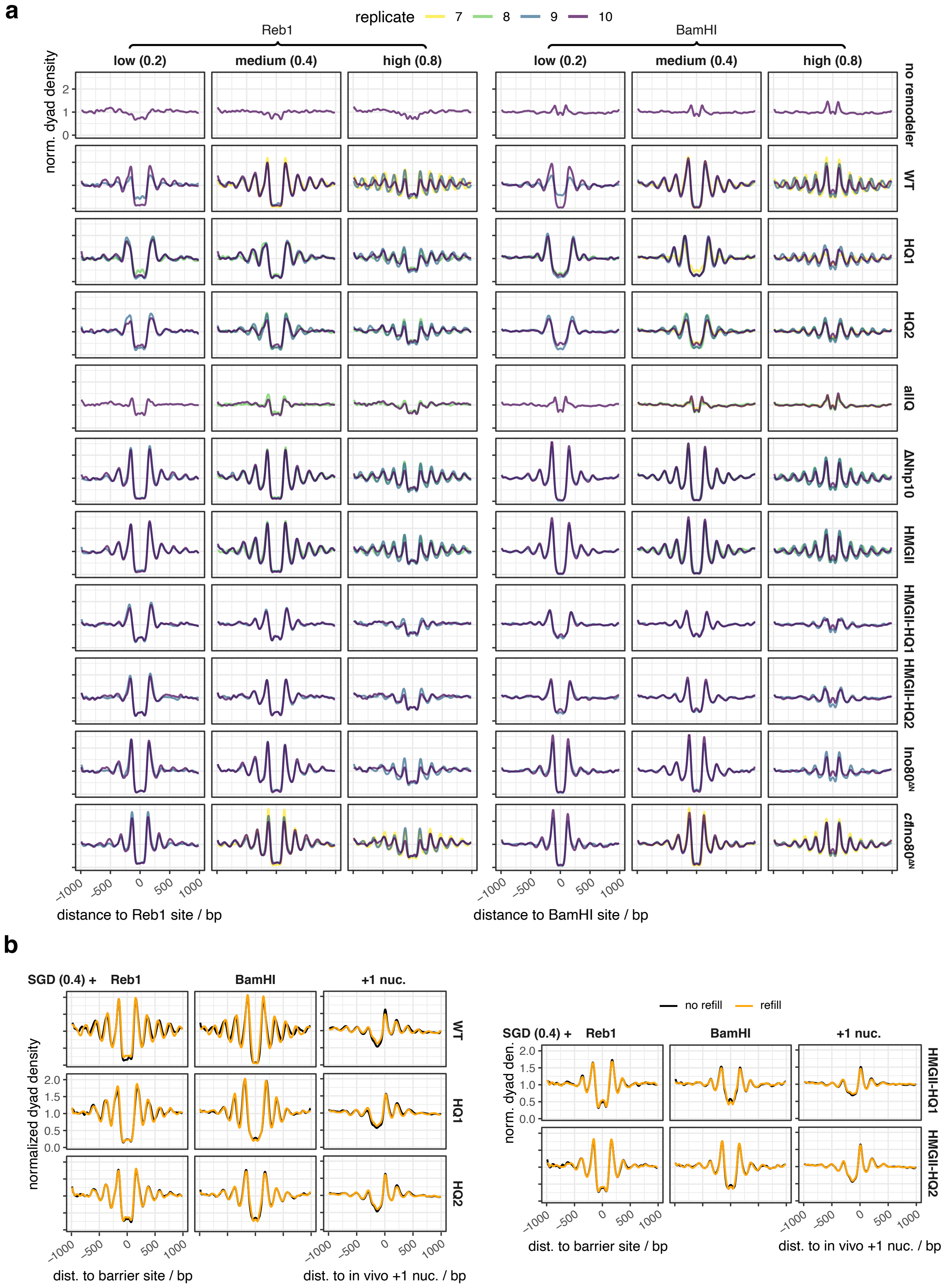

Supplementary Figure 5. **Plots of individual replicates for samples with WT and mutant INO80 complexes. a** As Supplementary Fig. 2c, but for the indicated WT and mutant INO80 remodelers and the *C. thermophilum* INO80 core complex (*ct*INO80$^{\Delta N}$). **b** As Supplementary Fig. 1c, but for indicated remodelers, SGD with medium assembly degree (SGD (0.4), single replicate) and alignment at in vivo +1 nucleosomes shown on the right of both subpanels.

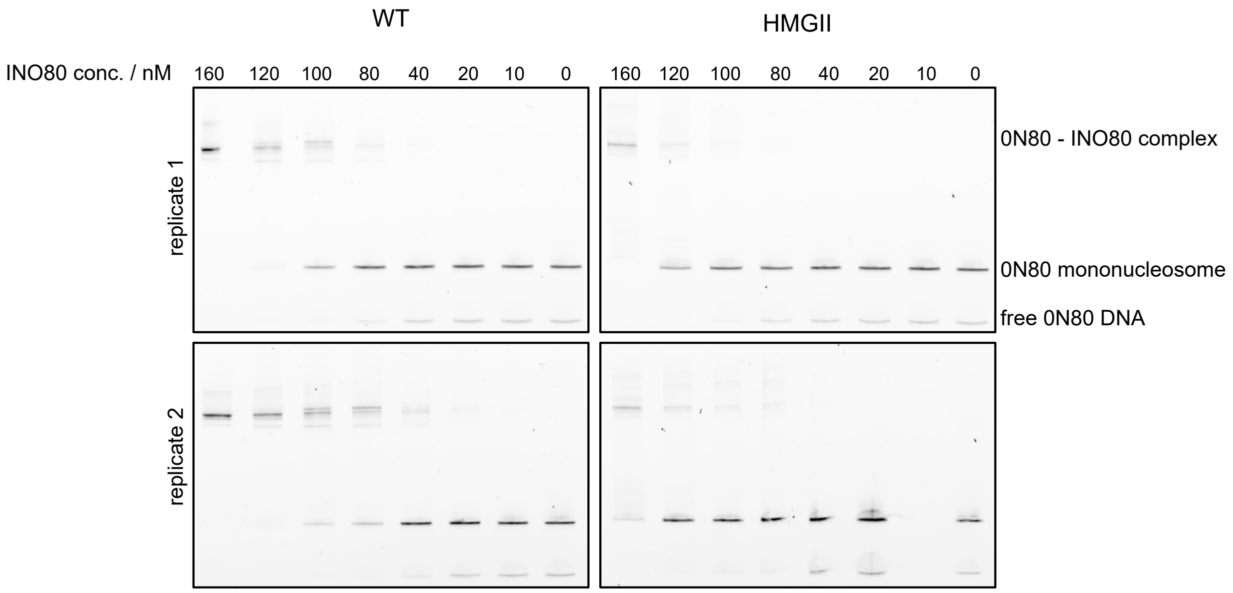

Supplementary Figure 6. **Reduced affinity for mononucleosome binding by the HMGII versus the WT INO80 complex.** Two replicates of gel retardation assays with the indicated concentrations of recombinant WT or HMGII mutant INO80 complexes and 20 nM mononucleosomes where a nucleosome reconstituted on the Widom 601 positioning sequence was flanked by no linker DNA on one side and 80 bp linker DNA on the other side (0N80 mononucleosome).

Supplementary Table 1. **Plasmids and primers used.**

REAGENT or RESOURCE	SOURCE	IDENTIFIER
Recombinant DNA		
pET21_601	Daniela Rhodes	N/A
pFBDM	pFastBacDUAL Invitrogen[5]	Addgene 110738
pFBDM-Rvb1-Rvb2-Ies6-Arp5-Ino80_HQ1	ref. 6	N/A
pFBDM-Rvb1-Rvb2-Ies6-Arp5-Ino80_HQ2	ref. 6	N/A
pFBDM-Ies1-Ies3-Ies5-ΔNhp10	This work	N/A
pFBDM-Ies1-Ies3-Ies5-Nhp10(HMGII)	This work	N/A
pFBDM-Rvb1-Rvb2-Ies6-Arp5-Ino80ΔN	This work	N/A
pET21b	Novagen	Addgene 72327
pET21b-Reb1	This work	N/A
Sequence based Reagents		
Primer Ino80-HQ1 for	CAACACCTATACAACTATTTGG AAAGACATGGCTCAGC	Metabion
Primer Ino80-H1Q rev	CATAGCCTCCTTTTCAATCTTC TTCTTTAGATCTCTTTC	Metabion
Primer Ino80_H2Q for	CAACACCTATACAACTATTTGG AAAGACATGGCTCG	Metabion
Primer ino80_H2Q rev	CATAGCCTCCTTTTCAATTTGC TGCTGTAGATCCTG	Metabion
Primer Nhp10-HMG for	GGCCTCCTTCAAACAGGAACT ATTGACGAAGCCATTTC	Metabion
Primer Nhp10-HMG rev	CAGATCTCTTGTCACGTCCAG AGAGCCATTCTG	Metabion
Primer Ino80$^{\Delta N}$ for	GCCTACGTCGACATGGCCCGT GCTATCCAGAGGCATT	Metabion
Primer Ino80$^{\Delta N}$ rev	CTGGATAGCACGGGCCATGTC GACGTAGGCCTTTGAATTCCG	Metabion

Supplementary references

1 Gutin, J. *et al.* Fine-Resolution Mapping of TF Binding and Chromatin Interactions. *Cell Reports* **22**, 2797-2807, (2018).

2 Rossi, M. J., Lai, W. K. M. & Pugh, B. F. Genome-wide determinants of sequence-specific DNA binding of general regulatory factors. *Genome Research* **28**, 497-508, (2018).

3 Oberbeckmann, E. *et al.* Absolute nucleosome occupancy map for the *Saccharomyces cerevisiae* genome. *Genome Res.* **29**, 1996-2009, (2019).

4 Zhang, Z. *et al.* A packing mechanism for nucleosome organization reconstituted across a eukaryotic genome. *Science (New York, N.Y.)* **332**, 977-980, (2011).

5 Trowitzsch, S., Bieniossek, C., Nie, Y., Garzoni, F. & Berger, I. New baculovirus expression tools for recombinant protein complex production. *Journal of Structural Biology* **172**, 45-54, (2010).

6 Knoll, K. R. *et al.* The nuclear actin-containing Arp8 module is a linker DNA sensor driving INO80 chromatin remodeling. *Nature Structural & Molecular Biology* **25**, 823-832, (2018).
