# Supplementary material for: Ruler elements in chromatin remodelers set nucleosome array spacing and phasing
Source: Nat Commun. 2021 May 28;12:3232. doi: 10.1038/s41467-021-23015-0 (PMC8163753; doi:10.1038/s41467-021-23015-0)
Supplement: Supplementary file 2 — Description of Additional Supplementary Files [file 41467_2021_23015_MOESM2_ESM.pdf]

Description of Additional Supplementary Files

File Name: Supplementary Data 1
Description: Summary of experimental details for each reconstituted chromatin sample used in this study. Each replicate is based on an individually assembled SGD chromatin prepared on a different day. The processed data files can be downloaded at GEO (GSE140614).
