# Supplementary material for: Ruler elements in chromatin remodelers set nucleosome array spacing and phasing
Source: Nat Commun. 2021 May 28;12:3232. doi: 10.1038/s41467-021-23015-0 (PMC8163753; doi:10.1038/s41467-021-23015-0)
Supplement: Supplementary file 5 — Reporting Summary [file 41467_2021_23015_MOESM5_ESM.pdf]

nature research

Corresponding author(s): Philipp Korber, Sebastian Eustermann, Karl-Peter Hopfner

Last updated by author(s): Feb 17, 2020

Reporting Summary

Nature Research wishes to improve the reproducibility of the work that we publish. This form provides structure for consistency and transparency in reporting. For further information on Nature Research policies, see Authors & Referees and the Editorial Policy Checklist.

Statistics

For all statistical analyses, confirm that the following items are present in the figure legend, table legend, main text, or Methods section.

n/a	Confirmed	
☐	☒	The exact sample size (*n*) for each experimental group/condition, given as a discrete number and unit of measurement
☐	☒	A statement on whether measurements were taken from distinct samples or whether the same sample was measured repeatedly
☒	☐	The statistical test(s) used AND whether they are one- or two-sided<br>*Only common tests should be described solely by name; describe more complex techniques in the Methods section.*
☐	☒	A description of all covariates tested
☒	☐	A description of any assumptions or corrections, such as tests of normality and adjustment for multiple comparisons
☐	☒	A full description of the statistical parameters including central tendency (e.g. means) or other basic estimates (e.g. regression coefficient) AND variation (e.g. standard deviation) or associated estimates of uncertainty (e.g. confidence intervals)
☒	☐	For null hypothesis testing, the test statistic (e.g. *F*, *t*, *r*) with confidence intervals, effect sizes, degrees of freedom and *P* value noted<br>*Give P values as exact values whenever suitable.*
☒	☐	For Bayesian analysis, information on the choice of priors and Markov chain Monte Carlo settings
☒	☐	For hierarchical and complex designs, identification of the appropriate level for tests and full reporting of outcomes
☒	☐	Estimates of effect sizes (e.g. Cohen's *d*, Pearson's *r*), indicating how they were calculated

Our web collection on statistics for biologists contains articles on many of the points above.

Software and code

Policy information about availability of computer code

Data collection	Illumina HiSeq 1500 with software provided by the manufacturer
Data analysis	Bowtie: 0.12.8; R Base: 4.0.2; R Studio: 1.1.463; GenomicAlignments: 1.18.1, see Methods section for full details

For manuscripts utilizing custom algorithms or software that are central to the research but not yet described in published literature, software must be made available to editors/reviewers. We strongly encourage code deposition in a community repository (e.g. GitHub). See the Nature Research guidelines for submitting code & software for further information.

Data

Policy information about availability of data

All manuscripts must include a data availability statement. This statement should provide the following information, where applicable:

- Accession codes, unique identifiers, or web links for publicly available datasets
- A list of figures that have associated raw data
- A description of any restrictions on data availability

All raw and processed sequencing data generated in this study were submitted to the NCBI Gene Expression Omnibus (GEO; https://www.ncbi.nlm.nih.gov/geo/) under accession number GSE140614.

Field-specific reporting

Please select the one below that is the best fit for your research. If you are not sure, read the appropriate sections before making your selection.

☒ Life sciences ☐ Behavioural & social sciences ☐ Ecological, evolutionary & environmental sciences

Life sciences study design

All studies must disclose on these points even when the disclosure is negative.

Sample size	Sample size equals replicate number stated for each experiment in main or supplementary figures and always in sample description linked to GEO deposition. No sample size calculation was performed due to exploratory nature of the research. Sample sizes were sufficient to reproduce our observations.
Data exclusions	No data were excluded.
Replication	In vitro reconstitutions were replicated with independent SGD chromatin preparations as detailed in statement of replicates (stated for each experiment in main or supplementary figures and always in sample description linked to GEO deposition). Apart from technical failures, all attempts at replication were successful .
Randomization	Randomization was not done as experimentator needed to know experimental conditions for conducting the biochemical experiments as well as for data analysis.
Blinding	Blinding was not done as experimentator needed to know experimental conditions for conducting the biochemical experiments as well as data analysis.

Reporting for specific materials, systems and methods

We require information from authors about some types of materials, experimental systems and methods used in many studies. Here, indicate whether each material, system or method listed is relevant to your study. If you are not sure if a list item applies to your research, read the appropriate section before selecting a response.

Materials & experimental systems

n/a	Involved in the study	
☐	☒	Antibodies
☐	☒	Eukaryotic cell lines
☒	☐	Palaeontology
☐	☒	Animals and other organisms
☒	☐	Human research participants
☒	☐	Clinical data

Methods

n/a	Involved in the study	
☒	☐	ChIP-seq
☒	☐	Flow cytometry
☒	☐	MRI-based neuroimaging

Antibodies

Antibodies used	Undiluted ANTI-FLAG® M2-Affinity Gel (product number A2220, Sigma Aldrich) was used for protein purification.
Validation	ANTI-FLAG® M2-Affinity Gel (product number A2220, Sigma Aldrich) was used for purification of remodelers and required therefore no additional validation.

Eukaryotic cell lines

Policy information about cell lines

Cell line source(s)	Spodoptera frugipeda Sf21 insect cells (Invitrogen; 11497013) used for INO80 wildtype and mutant virus production.<br>Trichoplusiani High Five insect cells (Invitrogen; B85502) used for INO80 wildtype and mutant expression.<br>Chd1 and FACT were expressed in Trichoplusiani insect cells.<br>Spodoptera frugiperda sf21 insect cells were used for virus production.<br>For purification of recombinant INO80 complex, CHD1 and FACT High Five (Hi5) insect cells (BTI-TN-5B1-4 Invitrogen) were used.
Authentication	Insect cell lines were purchased from Invitrogen (SF21: Invitrogen 11497013; Hi5: Invitrogen B85502) and used for Baculovirus-mediated protein expression without further authentication.
Mycoplasma contamination	Insect cell lines used for protein expression were not tested.
Commonly misidentified lines<br>(See ICLAC register)	No commonly misidentified cell lines were used in the study.

Animals and other organisms

Policy information about studies involving animals; ARRIVE guidelines recommended for reporting animal research

Laboratory animals	The Drosophila embryo histones were prepared from the Drosophila melanogaster strain OregonR.
Wild animals	The study did not involve wild animals.
Field-collected samples	The study did not involve field-collected samples.
Ethics oversight	No ethical approval was required as study only involved basic research with standard model organisms and cell lines.

Note that full information on the approval of the study protocol must also be provided in the manuscript.
